# Does updating education curricula accelerate technology adoption in the workplace? Evidence from dual vocational education and training curricula in Switzerland

**DOI:** 10.1007/s10961-022-09971-9

**Published:** 2022-11-28

**Authors:** Tobias Schultheiss, Uschi Backes-Gellner

**Affiliations:** University of Zurich, Plattenstrasse 14, CH-8032 Zurich, Switzerland

**Keywords:** Technology diffusion, Technology adoption, SMEs, Text as data, Innovation policy, Education curricula, O33, I25, J23

## Abstract

In an environment of accelerating technological change and increasing digitalization, firms need to adopt new technologies faster than ever before to stay competitive. This paper examines whether updates of education curricula help to bring new technologies faster into firms’ workplaces. We study technology changes and curriculum updates from an early wave of digitalization (i.e., computer-numerically controlled machinery, computer-aided design, and desktop publishing software). We take a text-as-data approach and tap into two novel data sources to measure change in educational content and the use of technology at the workplace: first, vocational education curricula and, second, firms’ job advertisements. To examine the causal effects of adding new technology skills to curricula on the diffusion of these technologies in firms’ workplaces (measured by job advertisements), we use an event study design. Our results show that curriculum updates substantially shorten the time it takes for new technologies to arrive in firms’ workplaces, especially for mainstream firms.

## Introduction

Even though accelerated technology diffusion is economically beneficial and particularly helpful for strengthening competitiveness in a digital economy (Bloom et al., 2013; Bosio & Cristini, 2018; Giorcelli, 2019), new technologies tend to flow too slowly from the innovation frontier into the mainstream of firms (Comin & Hobijn, 2010; O’Mahony & Vecchi, 2009; Syverson, 2011). While firms at the innovation frontier (hereafter, “frontier firms”) possess innovative knowledge on emerging technologies in-house (Andrews et al., 2015), firms farther away from the innovation frontier often struggle to keep up with new technologies (we call them “mainstream firms” to contrast them with “frontier firms”). Such mainstream firms often lack the necessary absorptive capacity because they, for example, do not have adequately trained workers (Cohen & Levinthal, 1989, 1990; Freel, 2005; Gritti & Leoni, 2012; Vinding, 2004). National innovation policies therefore often aim to boost the absorptive capacity of mainstream firms (Albors et al., 2006; Caiazza, 2016; Cassiman & Veugelers, 2006; Geroski, 2000), frequently by policies in the education system (Biasi & Ma, 2021; Goldin & Katz, 2008; Squicciarini, 2020).

However, previous research on the role of the education system has largely neglected the special role of updating education curricula to strengthen the absorptive capacity of firms. We close this gap by investigating curriculum updates for vocational education and training (VET) programs and their role in diffusing new technologies faster. We show that systematic and future-oriented curriculum updates can be an important lever of education-related innovation policies. Our contribution is important because our results demonstrate that the education system can strongly contribute to accelerating the diffusion of new technologies into mainstream firms by systematic and timely curriculum updates.

We exploit largely untapped text data from nationally binding training curricula and examine a setting in which curriculum updates are developed with the help of innovative firms. In particular, we study updates of Swiss dual VET curricula and how they affect technology diffusion into firms’ workplaces. The VET programs cover the training for almost all middle-skill occupations in the Swiss economy and thus define education and training content for about two-thirds of Swiss adolescents (State Secretariat for Education, Research and Innovation, 2019). About 80% of the training time takes place in firms, where apprentices work in regular production contexts. The curricula describe the training content not only for school part of the programs but also for this workplace part in high detail (26 pages on average). Curricula are jointly updated by actors from the public education sector, from (frontier) firms, and from industry associations. Particularly, frontier firms provide knowledge on the skills that will be required in an occupation’s future (Backes-Gellner, 1996; Pedró et al., 2009), based on the knowledge from their own R&D efforts (Rupietta & Backes-Gellner, 2019).

As soon as curriculum updates are legally enacted, the new curricula become legally binding and establish the foundations for training the new skills in a ready-made fashion (including training modules and inter-firm training courses as well as courses for instructors) for all training firms. Curriculum updates thereby (a) strengthen the absorptive capacity and heighten the technological awareness of firms by the time the new curricula are introduced and (b) increase the supply of workers with future-oriented skills after the first apprentices graduate from the updated programs. We hypothesize that through these two channels curriculum updates accelerate the diffusion of new technologies, particularly into mainstream firms.

To test our hypotheses, we need to overcome two major challenges. First, to establish a causal link, we need to overcome endogeneity problems. We do so by using an event study design that constructs the natural diffusion of new technologies (i.e., an unaffected baseline) and by exploiting the different timing of technology introductions across occupational curricula. We examine three key technologies from an early wave of digitalization: computer-numerically controlled (CNC) machinery, computer-aided design (CAD), and desktop-publishing (DP) software. These technologies are both impactful and well identifiable. To capture the natural diffusion trend that is exogenous to Swiss training firms, we use international patent data to capture when and how quickly these digital technologies emerged at the R&D-stage globally (i.e., outside and independently of Switzerland).

Second, we need to capture (a) when curricula received a technology update and (b) when—after the update—the new technologies began to arrive in the workplace. To measure the content of curriculum updates, we use previously untapped text data. While other studies using text data draw on, for example, texts from professional biographies (Fini et al., 2022) or patent texts (Webb, 2019), we draw on text data from curricula because we are interested in changes in the education system (similar to, e.g., Biasi & Ma, 2021; Droll et al., 2017; Fini et al., 2018; Gentzkow et al., 2019; George et al, 2016). Furthermore, to capture when technologies came into increasing use in workplaces of regular Swiss “production jobs” (i.e., jobs in the production of a firm’s goods or services as opposed to jobs in the firm’s R&D department), we use job advertisement texts from the Swiss Job Market Monitor (SJMM) (Buchmann et al., 2017).

Our results reveal that curriculum updates substantially accelerate the technology diffusion into the workplace. We find that, firstly, technology diffusion already accelerates during the implementation phase of a new curriculum, i.e., before apprentices even graduate and work as regular workers. Thus, curriculum updates already increase technological awareness and absorptive capacity through signaling the importance of a new technology in the implementation phase. Secondly, we find that technology diffusion further accelerates as soon as the first cohort of apprentices graduates after four years and becomes available as skilled workers. The increased supply of workers with future-oriented skills drives this second acceleration in technology diffusion.

## Theoretical framework

Absorptive capacity plays a crucial role in accelerating technology adoption and boosting the competitiveness of firms (Cohen & Levinthal, Freel, 2005; Su et al., 2013; Vinding, 2004; Volberda et al., 2010). Zahra and George (2002) define absorptive capacity as a “dynamic capability pertaining to knowledge creation and utilization that enhances a firm's ability to gain and sustain a competitive advantage.” Empirical studies on absorptive capacity find that absorptive capacity and innovativeness are tightly linked (Cepeda‐Carrion et al., 2012; Patterson & Ambrosini, 2015; Tzokas et al., 2015).

However, empirical studies also show that firms are very different in their absorptive capacity (Martínez-Román & Romero, 2017). Firms internalize innovative knowledge and adopt new technologies at very different speeds (Spithoven & Teirlinck, 2015; Veugelers, 1997). While some firms can gain a competitive advantage by faster technology adoption (Aboelmaged & Hashem, 2019; Corso et al., 2003; Levy et al., 2001; Nguyen, 2009), certain types of firms lack the absorptive capacity to internalize the knowledge on new technologies with the required speed.

Especially small and medium-sized enterprises (SMEs) often struggle to acquire and assimilate knowledge on new technologies fast enough, particularly knowledge relating to new digital technologies (Acar et al., 2005; Mole et al., 2004; Nguyen, 2009; Premkumar, 2003; Shin, 2006; Southern & Tilley, 2000). For CAD and CNC technology, Astebro (2002) shows that, in the U.S. metalworking industry, company size is one of the most important explanatory factors for the adoption of these technologies. However, strengthening the absorptive capacity can be an effective way to spur the adoption of such new technologies by SMEs (Love & Roper, 2015). In a study of more than 1500 SME owners across the U.K., Gray (2006) finds that absorptive capacity in SMEs facilitates the adoption of innovative technologies and subsequent growth of firms. Based on a sample of 2396 Spanish manufacturing firms, Gomez and Vargas (2009) show that absorptive capacity (as measured by internal R&D) is a strong predictor for the adoption of CAD, CNC, and robotics. Cozza and Zanfei (2016) find for Italy that firms organized within groups and networks—similar to an ecosystem—exhibit a greater propensity to access, absorb, and utilize external innovative knowledge.

Research also shows that a key factor for strengthening a firm’s absorptive capacity is having an adequately skilled workforce with the knowledge and skills needed for the new technologies (Ballantine et al., 1998; Bhagwat & Sharma, 2007; Bruque & Moyano, 2007). Vinding (2004) demonstrates, based on Danish data, that a highly skilled workforce positively impacts firms’ innovativeness. Freel (2005) finds that the innovativeness of SMEs and the skill level of their workforce are positively linked.

Another strand of literature shows that the education system plays an important role in providing the necessary skills in the “race between education and technology,” in which the education system can facilitate the use of new technologies (Autor et al., 2020a, 2020b; Goldin & Katz, 2008). For example, Beaudry et al. (2010) demonstrate that higher local skill supply (measured by education levels) led to an increased local adoption of the personal computer. In contrast, Lewis (2011) shows that an abundance of workers with low education levels slowed down technology adoption in U.S. manufacturing firms. Furthermore, a small of number of recent studies already indicate that updating education curricula can increase the access to innovative knowledge and improve the technical skills of the workforce (Biasi & Ma, 2021; Holzmann et al., 2020; Janssen & Mohrenweiser, 2018).

Combining the theory of absorptive capacity with the empirical findings from the literature on education systems for our setting of Swiss VET curricula, we expect curriculum updates to positively influence the adoption of new technologies mainly through two channels: first, curriculum updates strengthen firms’ absorptive capacity by increasing technological awareness and improving the knowledge base on new technologies of the firms’ current workforce. Instructors (for the workplace training part of the VET program) and apprentices bring the knowledge on new technologies into their firms immediately after the update and enable firms to internalize innovative knowledge on new technologies faster. One reason is that curriculum updates entail instructor courses, which give detailed guidance on the skills for new technologies and how to teach them. Another reason is that inter-firm training centers provide apprentices with the opportunity to acquire skills and obtain hands-on knowledge for new technologies (e.g., expensive machinery). Thus, apprentices are able to use the newest technologies even if these technologies are not yet available in their training firm. Furthermore, absorptive capacity may also improve during the training phase because curriculum updates may enhance awareness for new technologies and credibly signal the arrival of new technologies for firms along the whole production chain. In other words, curriculum updates make firms more aware that the industry is set for a change and that, for example, suppliers also gain from adopting new technologies. Thus, we expect the increased technological awareness and the newly acquired knowledge of apprentices and instructors to be the first channel through which curriculum updates boost the absorptive capacity of firms and subsequently affect technology adoption.

A second channel through which curriculum updates accelerate the adoption of new technologies is the greater availability of workers with new skills on the internal or external labor market. This effect sets in once the first graduates complete their updated training programs. The increased skill supply gives all firms easier access to the skills they need to adopt new technologies, which consequently decreases the cost of technology adoption and increases the speed at which firms are able to absorb the knowledge on new technologies.

## Institutional features of VET curriculum updates and data sources

Understanding some of the institutional features of the Swiss VET system in more detail is crucial for understanding the ways in which we exploit our data sources, solve endogeneity problems, and arrive at our findings and causal conclusions. The updating of the Swiss VET curricula offers a unique setting for examining the link between education and technology for four reasons: first, VET curricula are representative, in that they determine the training for roughly two-thirds of the Swiss workforce in approx. 220 occupations (State Secretariat for Education, Research and Innovation, 2019). The updating of VET curricula therefore translates into substantial changes in the skills of the Swiss workforce.^1^ Second, VET curricula codify the education and training content of each VET occupation at the national level, contain extensive skill information and training instructions, and are legally binding for all training providers. Third, final examinations covering the entire curriculum content ensure that VET graduates have actually obtained the specified skills from the curricula, including the new technology skills (Oswald-Egg & Renold, 2015). Fourth, curricula are regularly updated according to a legally mandated and well-structured process, which strongly involves innovative firms and takes place in regular intervals, with timing unrelated to technological factors (Backes-Gellner, 1996; Rupietta & Backes-Gellner, 2019). We explain the institutional features of the curriculum updating process that are critical to our empirical approach in more detail in the following subsection.

### Institutional features of the updating process

The VET curriculum development and updating system constitutes one part of the broader Swiss innovation ecosystem. Within such innovation ecosystems, well-defined institutional processes play an important role for organizing inter-firm knowledge exchange (Benny Carlsson & Stankiewicz, 1991; Freeman, 1988; Justman & Teubal, 1995; Watkins et al., 2015b). The updating process for Swiss VET curricula is institutionalized at the national level and serves as an important cornerstone for facilitating the exchange of knowledge on new technologies and skills across the whole Swiss innovation ecosystem as described in the following.

One key feature of the curriculum updating process is the strong connection between employers and the education system, that is, the active participation of innovative firms in the updating process (Bolli et al., 2018). In the updating process, multiple actors—industry associations, trade unions, professional associations, innovative firms, and government organizations—collectively gather information on new trends in the industry and in the workplaces of the respective occupation and then update the existing apprenticeship training curriculum. (To better illustrate the updating process, the Appendix provides case studies and anecdotal evidence from several industries, based on Backes-Gellner & Pfister, 2019).^2^

The updating process is *cyclical* (i.e., it occurs in regular intervals), *structured* (i.e., the coordination of the actors follows internal procedures), and *future oriented* (i.e., the content update takes new technological or organizational developments and the most recent innovation trends for the respective workplaces into account). Industry associations and innovative firms deliver inputs for the new training content and updating of curricula. Moreover, they help to prepare new training material for the instructors at the training firms. Government organizations supervise the legal and regulatory aspects of the process (such as enacting the new ordinances) and assist with the implementation of the new training requirements in VET schools and in final examinations.

The exact timings of the enactment of new curricula are largely driven by technology-unrelated regulatory and administrative factors of the curriculum updating process. These regulatory factors include the requirement to fully review curricula and evaluate their need for updates within fixed timeframes, which are set independently of any particular technology trend.^3^ These unrelated factors also include delays by regulatory authorities, which represent a regulatory bottleneck to the enactment of a new curriculum and depending on authorities’ workload (unrelated to a particular technology trend) lead to a considerably faster or slower approval of an updated curriculum. Moreover, from the perspective of most firms (especially SMEs), curriculum updating is an exogenous event because SMEs are too small to influence when the occupation-wide updating will take place.

Our paper examines whether new technologies become part of mainstream workplaces faster in the years following curriculum updates introducing the respective technology skills. To empirically capture this diffusion effect of updates, we require data on (1) when new technology skills were introduced into curricula, (2) when and how quickly the underlying new technologies emerged at the R&D stage, setting a baseline for technology diffusion, and (3) when new technologies came into increasing use in the workplace.

### Timing and content of VET curriculum updates

To determine when and for which occupations new technology skills became part of the curricula, we self-collected information on the timing of the curriculum updates and the changes in content. We focus on our set of well-identifiable digital technologies, that, is CAD, CNC, and DP. For the timing of the updates, we use information from the Swiss Database for Occupational Development (“Datenbank Berufsentwicklung”), which documents the evolution of each of the approximately 220 Swiss VET occupations since 1931. Education in each VET occupation follows its own occupational curriculum: the database allows us to identify when an occupation received an update, was merged into another occupation, or changed names.^4^ Using this information, we follow an occupation and all its predecessor occupations over time and determine the years in which they were updated. In addition to the year of updating, we determine the year in which the first apprentices graduated under the updated curricula and entered regular jobs.

To capture the new content added by the updates, we analyze the actual texts before and after the updating. The skill descriptions from the curricula constitute official government documents (“Reglemente,” “Bildungsverordnungen,” and “Bildungspläne”) that are published in the Bulletin of the Swiss Confederation (“Bundesanzeiger”) and are thus very well documented. We use the text as data and take a quantitative text analysis approach to capture the changes in the curriculum content, that is, whether and, if so, when one of our technologies was introduced in an updated VET occupation.

The curriculum texts contain information on the organizational structure (e.g., the length of the apprenticeship), the requirements for firms hiring apprentices (e.g., educational requirements for instructors), the learning objectives (e.g., the skills and techniques that an apprentice must have acquired by the end of the second year), and the teaching content (e.g., how many lessons on specific technical subjects are needed). We use keywords identifying our technologies (e.g., “CAD” or “computer-aided design”) and search for their appearance in the curricula. We also take into account the historical names and German terms for our technologies (e.g., “computer-gestütztes Konstruieren”). When these terms clearly appear in an updated curriculum text, we consider this curriculum to be updated with that technology.

As each of the about 220 occupations follows its own occupational curriculum, the occupations represent the level of educational intervention. Several occupations are affected by our technologies (Table 6 in the Appendix reveals the staggered introduction of the technologies across these occupations): various drafter occupations (affected by CAD technologies), engineering and carpentry occupations (affected by CNC technologies), and design and media occupations (affected by DP technologies). However, to measure the acceleration in technology diffusion beyond its natural rate, we first need to establish a baseline for the natural diffusion of CAD, CNC, and DP. We achieve this by drawing on the global R&D-stage diffusion of these technologies.

### Technology diffusion at R&D stage

To measure when and how quickly a new technology emerged at the R&D stage, we draw on patent data from Scopus. In this approach, we follow previous studies that use innovation data (i.e., patents) to measure R&D activities (Griliches, 1998; Lybbert & Zolas, 2014) and the emergence of new technologies (Kogan et al., 2021; Webb, 2019). A growing number of patents indicates that innovative firms (based on their own R&D efforts) are developing new technologies and applying them to new production processes or products (Calel & Dechezleprêtre, 2016; Campbell, 1983; Fu et al., 2018; Guellec & van Pottelsberghe de Potterie, 2001).

We focus on our set of well-identifiable technologies (CAD, CNC, and DP), which constitute key technologies from an early wave of digitalization that took place from the late 1980s to the early 2000s. Today these technologies are commonly used in all industrialized countries. These technologies emerged as global phenomena at the R&D stage and became naturally more widespread in their application in frontier firms, i.e., those with R&D departments and located at the innovation frontier. To capture this innovative capacity in our technologies, we calculate the cumulative global number of patents for each technology. The cumulative number of patents represents the stock of knowledge in a technology, produced by R&D and ready to be applied (as patents reflect the application of new knowledge, we later complement it with an additional control for basic research, i.e., research papers). The cumulative patents vary over time and between our technologies of interest (with the maximum number of patents in 2019 being for CAD, with 77,730 cumulative patents, and the minimum being for DP, with 6999 cumulative patents).

To account for these level differences in patents across technologies, we take the cumulative number of patents (i.e., the patent stock) and use it as an index with 2019 as the base year (i.e., we set the 2019 patent number to 100 for each technology separately). This procedure ensures that measuring the R&D-stage diffusion across our three different technologies is comparable and reflects the knowledge stock at the R&D stage in each of our three technologies in a certain year.

**Fig. 1:**
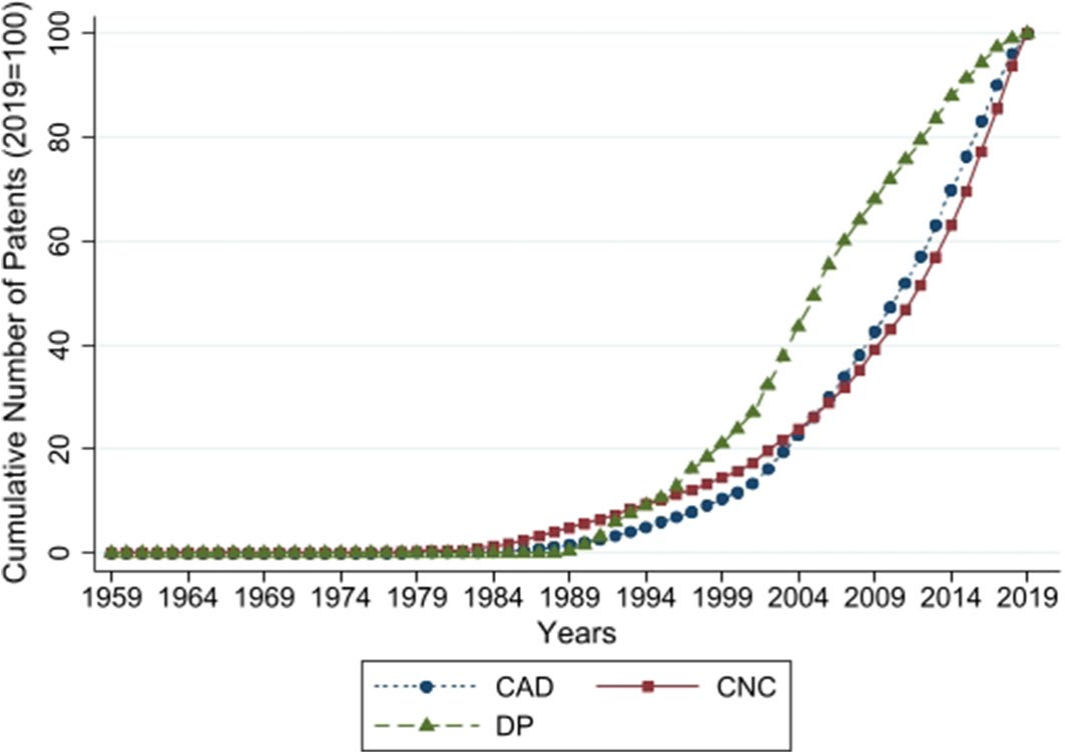
Technology diffusion at the R&D stage (Measured by the cumulative number of patents for each technology, as an index with 2019 as base year = 100). *Notes* Cumulative number of patents refers to an index of the yearly cumulative sum of patents, with 2019 as base year. Authors’ calculations with data from Scopus

Figure 1 shows the cumulative number of patents for our technologies, capturing when and how quickly the knowledge on the application of the new technologies diffused at the R&D stage. All of our technologies started to emerge in the mid-1980s, with DP showing the fastest rate of R&D-stage diffusion, which began in the 1990s, compared to CAD and CNC. The R&D-stage diffusion enables us to capture the spread of applied knowledge and establish a baseline of how quickly these technologies diffuse naturally at a global level. As patents reflect mainly the application of new knowledge, our empirical approach also uses a similarly constructed control variable for basic research based on the number of research papers in each of our technologies (CAD, CNC, and DP). This additional control variable then complements our R&D-stage diffusion measure, assuming that the frontier of knowledge in basic research and applied research move at different speeds and both influence technology diffusion. As the next step for capturing the diffusion effects of curriculum updates, we need to capture how quickly these technologies spread into the production jobs of Swiss firms.^5^

### Technology use in production jobs

To precisely measure technology use and its diffusion into production jobs (as opposed to R&D jobs), we use job ad data. The descriptions of job ads capture actual job content (Marinescu & Wolthoff, 2019) and contain information on the technology that workers use and the required skills for doing so (Deming & Noray, 2020; Hershbein & Kahn, 2018). As including additional words in job ads incurs a substantial extra cost (particularly in the period that we study), firms mention technologies only if they are relevant to the open job position and already in use at the workplace.

We draw on data from the SJMM, which is based on a representative sample of job ads for Switzerland from 1950 to 2019. The SJMM encompasses job ads for regular jobs and contains the full text for the sampled job ads in preprocessed forms, offering high-quality text data. As the SJMM team manually typed and coded the job ads for their own research purposes (Buchmann et al., 2017), the SJMM data has no missing word elements and contains rich information on the characteristics of each job ad. These characteristics include the required educational level and occupational training, the main task of the job position, the industry of the firm with the job vacancy and the firm’s size, the advertisement channel (newspaper, firm website, or job portal), and the workplace location. In Switzerland occupations and occupational training are highly standardized and, therefore, firms commonly and explicitly state the required formal occupational qualification in their job ads (e.g., “we are looking for an apprenticeship graduate in Polymechanics”).^6^ The explicit mentioning enables us to precisely capture these occupational requirements.

As we are interested in the arrival of new technologies in the mainstream, we focus on the jobs in the production of goods and services as opposed to the jobs in R&D, which are situated at the innovation frontier.^7^ We use the main-task classification to exclude the job ads that are for jobs in R&D and concentrate on the remainder of the ads, i.e., the ads for production jobs.^8^

For these job ads, we dig deeper into the job ad texts. We use a classification that assigns each word of the job ad text to one of eight categories, called “textzones” (based on Gnehm, 2018). Taken together, the textzones mimic the typical job ad structure, such as descriptions of both the firm and the vacant job position and the required hard and soft skills. After lemmatization (i.e., reducing words to their dictionary form) and removing stop words (e.g., “at,” “which,” and “and”), these textzones contain sets of keywords. To determine whether the job positions involve the use of one of our technologies, we search the textzones *firm description* (e.g., “our firm operates with the newest CNC machines”), *job description* (e.g., “you will have CAD systems at your disposal”), and *hard skills* (e.g., “knowledge of desktop publishing software required”) for keywords that clearly and unambiguously identify our technologies. For example, to identify CAD, we search the respective textzones of each job ad for the appearance of “CAD” (an abbreviation also used commonly in job ads in German) and “computer-aided design.” To ensure that we do not pick up merely changing vocabulary trends for the technology, we augment our search with the German historical equivalents of the English terms (e.g., “computer-gestütztes Konstruieren”) as well as the keywords for specific software (e.g., “ABViewer”). In our approach, we follow Hershbein and Kahn (2018) and Atalay et al. (2018), who take a keyword approach to identify technologies in job ads and thereby technology use in the workplace.

If the job ad contains at least one keyword representing our technologies,^9^ our outcome variable *technology use* takes the value 1 (i.e., this job ad mentions the technology); it takes the value 0 otherwise.^10^

**Fig. 2:**
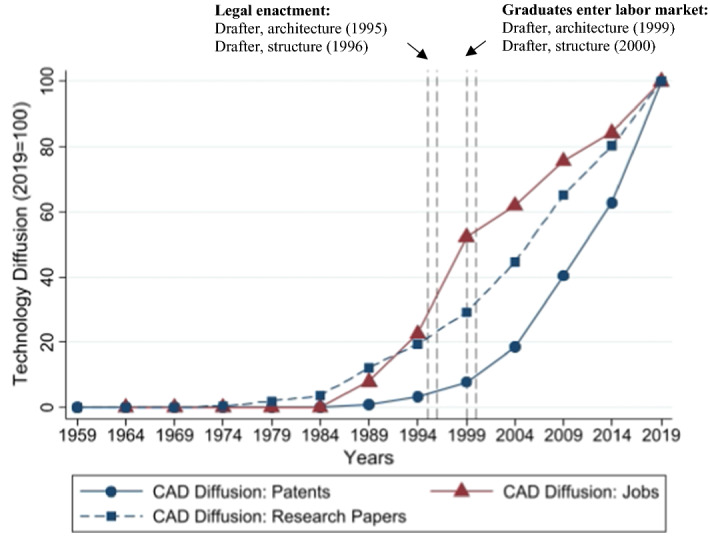
Technology diffusion into jobs of selected draftsman occupations (as Measured by Job Ads). *Notes* Average percentage of technology use for drafters in architecture and structure, in 5-year bins and as an index with 2015–2019 as base. CAD Diffusion: Patents refers to the index of cumulative patents in CAD in 5-year bins and 2015–2019 as base. We additionally report CAD Diffusion: Research Papers, which is an index of cumulative research papers in CAD in 5-year bins and 2015–2019 as base. Authors’ calculations with data from the SJMM

To assess whether curriculum updates accelerate technology use beyond its natural diffusion, we link the job ads by their year and occupational requirements to the timing of the curriculum updates and to the level of R&D-stage diffusion. Combining the three data sources then enables us to empirically capture the diffusion effect of curriculum updates. Table 1 displays summary statistics for all variables that we extracted from our data sources, while Table 2 shows with the help of a correlation matrix how these variables are correlated. Figure 2 illustrates—as an example—when and how quickly new technologies came into increasing use in two drafter occupations, architecture and structure (for a comprehensive overview of diffusion curves across all three technologies, see Fig. 6 in the Appendix). The graphs show that after the updates (in 1995 and 1996), the technology use in both occupations accelerated substantially in the short and medium run—and beyond the natural diffusion implied by patents (and research papers). After the updates, the diffusion curve becomes much steeper. To examine whether these patterns reflect a causal mechanism triggered by the updates and entail effect sizes that are economically relevant, we use an event study design.

**Table 1:** Summary statistics

	Obs	Mean	Std. Dev	Min	Max
*A. Diffusion measures*					
Technology use in jobs	4215	0.20	0.40	0	1
R&D-stage: patents (Absolute)	4215	11,535	18,375	0	77,730
R&D-stage: patents (Index)	4215	40.68	33.01	0	100
Research papers (Absolute)	4215	20,744	38,972	0	138,807
Research papers (Index)	4215	42.01	32.96	0	100
*B. Curriculum updates*					
Curriculum update	4215	0.66	0.47	0	1
*C. Firms’ characteristics*					
Small- and medium-sized firm	4215	0.84	0.37	0	1
Patent-applicant firm	4215	0.20	0.40	0	1
*D. Job advertisement characteristics*					
Number of words	4215	129.39	92.77	1	730
Newspaper as advertising channel	4215	0.42	0.49	0	1
Firm website as advertising channel	4215	0.38	0.49	0	1
Jobportal as advertising channel	4215	0.19	0.40	0	1

Sample consists of job ads for occupations affected by CAD, CNC, and DP (i.e., drafter occupations; mechanical engineering and carpentry occupations; and graphic design and media occupations) from 1980 to 2019. Authors’ calculations with data from the Swiss Job Market Monitor

**Table 2:** Correlation matrix

	1	2	3	4	5	6	7	8	9	10
1. Technology USe	1.00									
2. Patents (Index)	0.27^***^	1.00								
3. Papers (Index)	0.25^***^	0.99^***^	1.00							
4. Curriculum update	0.25^***^	0.72^***^	0.69^***^	1.00						
5. Small/Medium-sized firm	− 0.06^***^	− 0.03	− 0.03	− 0.11^***^	1.00					
6. Patent-applicant firm	0.00	− 0.04^**^	− 0.04^**^	− 0.03	− 0.17^***^	1.00				
7. Number of words	0.23^***^	0.58^***^	0.58^***^	0.48^***^	− 0.26^***^	0.11^***^	1.00			
8. Channel: newspaper	− 0.23^***^	− 0.76^***^	− 0.75^***^	− 0.74^***^	0.13^***^	0.03^*^	− 0.54^***^	1.00		
9. Channel: firm website	0.16^***^	0.46^***^	0.45^***^	0.50^***^	− 0.16^***^	0.01	0.29^***^	− 0.67^***^	1.00	
10. Channel: jobportal	0.10^***^	0.38^***^	0.38^***^	0.30^***^	0.02	− 0.06^***^	0.32^***^	− 0.42^***^	− 0.39^***^	1.00

Pearson correlation coefficient for our outcome of interest and all covariates. Sample consists of job ads for occupations affected by CAD, CNC, and DP (i.e., drafter occupations; mechanical engineering and carpentry occupations; and graphic design and media occupations) from 1980 to 2019. Significance levels: *p < 0.10, **p < 0.05, ***p < 0.01

## Event study design

An event study design enables us to estimate diffusion effects and to track these effects over time. Moreover, it allows us to investigate potential threats to identification. We use the following event study specification, with the individual job ad $i$ (posted for occupation $o$ with technology $h$ in year $t$) as the level of observation:

$$Technology\_Use_{i,o,h,t } = \alpha + \beta R\& D\_Diffusion_{h,t} + \sum\limits_{{j \in {\Phi }}} {\gamma_{j} Update_{o,t}^{j} + X_{i,o,h,t} + \upvarepsilon _{i,o,h,t} }$$

where ${Technology\_Use}_{i,o,h,t}$ denotes a binary variable taking the value of 1 if the job ad mentions technology $h$ (i.e., CAD for drafter occupations, CNC for engineering and carpentry occupations, and DP for design and media occupations) and 0 otherwise.^11^${R\&D\_Diffusion}_{h,t}$ uses the cumulative number of patents to capture the general diffusion at the R&D stage of the respective technology $h$ in year *t* in which the job ad was posted. With technology diffusion often following an s-shaped pattern (Geroski, 2000; Jovanovic & Lach, 1989), we include polynomials for the second and third order of the indexed cumulative number of patents.

To alleviate concerns about reverse causality, such as the Swiss educational system or the curriculum content affecting the number of patents in each year, we exclude patents from Switzerland and the European Patent Office (EPO) from ${R\&D\_Diffusion}_{h,t}$. The exclusion ensures that we construct the R&D-stage diffusion from an *independent* and *unaffected* data source. The R&D-stage diffusion reflects a worldwide trend, in which Switzerland, as a small economy (with a population of 8.6 million), does not play a significant role.

To model the natural diffusion trend of new technologies (i.e., the counterfactual diffusion trend without curriculum updates in Switzerland), we draw on this worldwide R&D-stage diffusion measure and use it as a benchmark.^12^ Given this research design, we expect to observe a positive diffusion effect when technology use in Swiss firms accelerates faster than expected according to the cumulative number of global patents (i.e., the level of R&D-stage diffusion).^13^ Our parametric approach (similar to Krupp & Pollard, 1996, or Siming, 2017) enables us to construct a proper counterfactual despite data constraints such as having highly asymmetric numbers of observations across occupations and a lack of occupations that could be potential receivers of a curriculum update but never receive it (i.e., never treated units).

The curriculum updates take place at the occupational level. We use the distance to the updating event as a way for studying the diffusion effects before and after curriculum updates. To reduce noise from short-term fluctuations in job ads, we employ two-year bins for the distance to the event: we use event indicators for each two-year bin in the six years before and the 10 years after the update.^14^ The treatment variables of interest are therefore a set of occupation-specific indicator variables ${Update}_{o,t}^{j}$ that take the value of 1 when the enactment of the occupational curriculum for occupation $o$ is $j$ years away, with $j\in\Phi$ and $\Phi = -6,-4, -2, ... 8$. We expect curriculum updates to have strong short- and medium-run effects, which reflect that updates bring new technologies into jobs faster, that is, by some years earlier, compared to firms in a counterfactual situation (i.e., industries/occupations without or with delayed updating). We also hypothesize that these effect taper off in the long run with large-scale technology adoption.

**Fig. 3:**
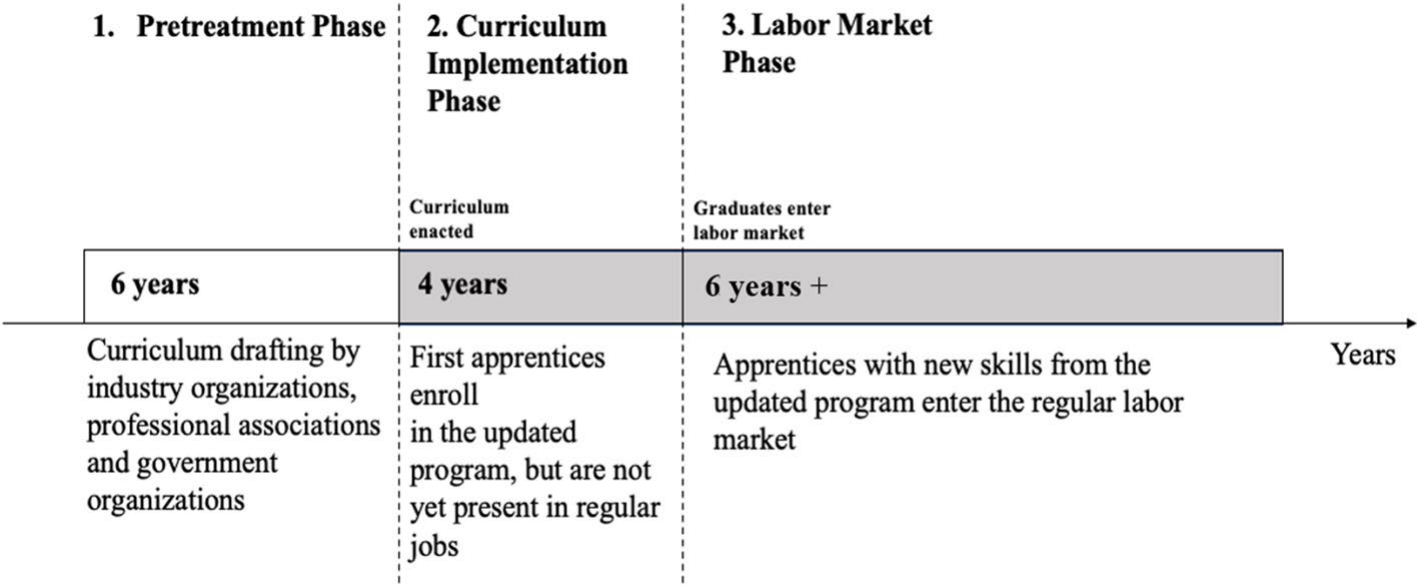
Time structure of curriculum updates. *Notes* The pretreatment phase is set to 6 years, because the drafting of a new curriculum update may take up to six years; see Backes-Gellner and Pfister (2019). The implementation phase is four years because all occupations in our sample involve four-year VET programs.

Figure 3 illustrates how these event indicators, which cover a span of 16 years around the year of the update, account for the typical curriculum updating process^15^:

1. *Pretreatment phase (6 years)* This phase encompasses the years in which the curriculum updating process has been launched but has not yet been completed. We set this pretreatment phase at six years because once a curriculum update has been officially scheduled, the process of determining which technical developments should be considered (and which new skills are therefore required), drafting new training contents, and legally enacting the new curriculum can take between four and six years (Backes-Gellner & Pfister, 2019). The coefficients ${\{\gamma }_{j}\}$ for $j=-6, -4, -2$ correspond to the precurriculum periods. They enable us to investigate whether the firms exhibit any anticipation effects of the coming intervention and to detect whether an upward trend in technology use antecedes legal updating. Both are potential threats to our identification strategy.
2. *Curriculum implementation phase (4 years)* This is the phase when firms begin training the first apprentices under the legally enacted new curricula but before the first apprentices graduate. Apprentices with updated skills are therefore not yet available as regular workers in the labor market. As the occupations in our sample all involve a training length of four years, the implementation phase spans four years. During this phase, the training of instructors via new training handbooks and instructor courses (to prepare them for teaching the new skills) and the training of apprentices in the new technology skills (also via inter-firm training courses) may already start to diffuse awareness and knowledge for new technologies.^16^ As apprentices progress in their apprenticeship programs, they are increasingly working on skilled tasks in regular production processes (a result found in many cost-benefit studies, e.g., in Moretti et al., 2019; Muehlemann & Wolter, 2014; Wolter et al., 2006) and thus likely become better acquainted with the new technologies. The coefficients ${\{\gamma }_{j}\}$ for $j=0, 2$ reflect these diffusion effects and reveal whether the new technologies come into the workplace through the previously mentioned channels.
3. *Labor market phase (six years)* This is the phase when the first apprentices graduate from an updated VET program, that is, when they are available as skilled workers on the labor market. The coefficients ${\{\gamma }_{j}\}$ for $j=4, 6, 8$ capture the diffusion effect from the increased supply of new technology skills in the labor market.

For optimal coverage of the diffusion and updating processes, we start our observation period in 1980 (our technologies first begin to appear in job ads in the early 1980s, with the first mention being CNC technologies in 1981), and we end the observation period in 2019, which is the latest data available from the SJMM.

${X}_{i,o,h,t}$ denotes a set of control variables (Table 3 gives an overview of all our variables and their definitions). Given the long observation period (1980–2019), potential confounders arise mainly from three domains: first, the way the job ads are written (i.e., the trend towards more online job ads with longer text and thus a higher chance of mentioning technologies); second, technological advances that are not covered by the cumulative number of patents; and, third, firm characteristics that affect technology use.

**Table 3:** Overview of variables included in the empirical approach

Class	Variable	Type of variable	Description	Data source
Dependent variable	Technology use	Binary	Indicator taking value 1 if the job ad mentions the use of new technology (CAD, CNC, DP)	Swiss job market monitor
Independent variables	Update	Binary	Set of event study indicators taking value 1 if curriculum is updated	Curricula, collected by authors
	R&D diffusion	Continuous	Index of cumulative number of patents (with 2019 as the base year)	Scopus
	Channel	Categorical	Channel that the job ad appeared in (newspaper, firm website, or online job board)	Swiss job market monitor
	#AdWords	Continuous	Number of words in the job ad	Swiss job market monitor
	Research papers	Continuous	Index of cumulative number of research papers (with 2019 as the base year)	Scopus
	Firm size	Categorical	Size of the firm (small and medium-sized firms versus large firms)	Swiss job market monitor
	Patent-applicant firm	Categorical	Firm as patent applicant (no patent applications versus patent applications in the past)	Swiss job market monitor/Google patents

Descriptions of variables, including their definitions and data sources

In our baseline specification, we use the characteristics of job advertisements available in the SJMM (similar to previous studies with job ads; e.g., Atalay et al., 2018). We adjust for the trend towards longer job ad texts by controlling for the number of words in the job ad. We also control for the channel in which the job ad appeared (i.e., whether it appeared in a newspaper, on a firm website, or an online job board) because the channel may also impact how the job ad is written and how long it is (e.g., by affecting the cost of including additional words in the job ad).

In our second specification, we also control for technological advances that might not be captured by our main measure for R&D-stage diffusion. Patents capture mainly the application of new knowledge to firms’ production processes (applied research) as opposed to the generation of new basic knowledge (basic research). As R&D-stage diffusion and advances in basic research on new technologies may move at different speeds and both likely affect technology adoption, we complement our patent-based measure with a similarly constructed measure for basic research. We include the cumulative number of research papers (as an index, with 2019 as the base year) into our regression.

In a third specification, we control for firm characteristics, in particular, those relating to firms’ in-house capacities to absorb knowledge on emerging technologies. To control for innovative or larger firms naturally having a higher absorptive capacity and adopting new technologies earlier, we include controls for these two firm characteristics in a third specification. In particular, we control for firm size, that is, whether the job ad is from a small or medium-sized enterprise (SME) (as opposed to a large firm). Moreover, we control for innovativeness, that is, whether the firm has filed patent applications in the past.

## Results: Technology diffusion effects of VET curriculum updates

### Main results: Technology diffusion

**Fig. 4:**
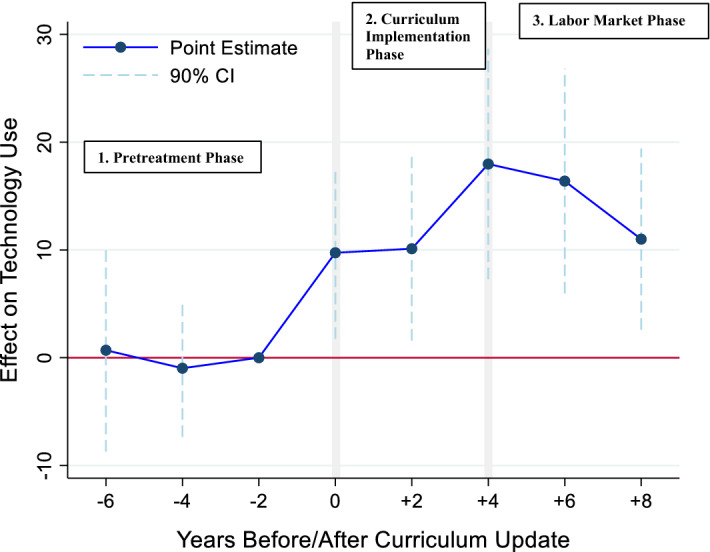
The Effect of Curriculum Updates on Technology Use on the Workplace (as Measured by Job Ads). *Notes* Results from the linear probability regression, following our baseline specification, which controls for R&D-stage diffusion, the number of words, and the advertisement channel of the job ad. Technology Use multiplied by 100 to represent percentage points. The two years before the enactment of a new curriculum serve as the reference category for the remaining event indicators and are therefore excluded. Event indicators cover two-year periods, e.g., 0 covers the year of the update and the year that follows. R&D-stage diffusion as an index with 2019 as base year; squared and tripled terms included. Standard errors based on the complex survey design by the SJMM.

Our event study results show a direct link between updating curricula and an accelerated technology diffusion: updates substantially shorten the time until new technologies arrive in more widespread use in production jobs. Figure 4 graphically summarizes the main results, with the x-axis denoting the years before (after) an occupation received its curriculum update. The y-axis shows the estimated effect of the curriculum update (in percentage points) on technology use in the workplace, i.e., on the probability that a job ad mentions the use of the new technology in the advertised job. The reference category for our event indicators are the two years before the enactment of the new curriculum. As we control for the R&D-stage diffusion, the estimated effects capture any change beyond the naturally occurring technology diffusion (from increased R&D activities). One way of interpreting the diffusion effects is that curriculum updates increase the steepness of the technology diffusion curve, bringing new technologies from the innovation frontier into production jobs sooner by a considerable number of years.

In the pretreatment phase (i.e., before a curriculum update), we find no significant effects for our event indicators. A joint F-test also reveals no significant differences with zero, that is, there is no indication of an unexplained upward trend in technology use in Swiss firms’ workplaces before the curriculum update. Before the updates, technology use in the workplace follows the naturally occurring trend implied by the global R&D-stage diffusion.

In the implementation phase, that is, with the implementation of a new curriculum, beginning with the second year of training under the updated curriculum, the update starts having an effect on technology use in mainstream workplaces in Switzerland. During the four years of the curriculum implementation phase, we find a substantial increase in mentions of technology use in the job ads of the updated occupations. The increase amounts to 10 percentage points beyond the natural diffusion implied by the R&D-stage diffusion and compared to not-yet-updated occupations.^17^ For the channel underlying this effect, we argue that curriculum updates begin to strengthen the absorptive capacity of firms right after the implementation and likely signal the arrival of new technologies to the broader industry.

In the labor market phase, i.e., when the first apprentices under the new curriculum graduate and enter the regular labor market (internal or external), we find that the diffusion effect becomes even stronger. The curriculum updates accelerate the technology diffusion by up to 18 percentage points beyond the natural diffusion and compared to the not-yet-updated occupations (estimation results with yearly event indicators can be found in Fig. 7 in the Appendix and show similar diffusion patterns). We attribute this diffusion effect to the increased skill supply, which allows firms to more easily complement the introduction of the new technologies with the needed skills on the worker side. This channel then substantially reduces the costs of adopting the new technologies and spurs technology adoption.

More broadly, we interpret these diffusion patterns as follows: exogenous changes in the education curricula cause changes in firms’ awareness of new technologies and in the available skill supply on the labor market—and, as a consequence, these changes then de facto transform the workplaces. Finding the strongest diffusion effects very closely after curriculum updates aligns with our expectation that updates substantially shorten the time until workplace transformation occurs through new technologies.

To better assess the economic importance of the curriculum intervention, we estimate the average effect over the entire period after the respective update. Column (1) in Table 4 reports the results from our baseline specification, showing an average diffusion effect of about 12 percentage points.^18^ When including controls for basic research (i.e., the number of research papers) for our second specification, the diffusion effect is only slightly lower (Column 2). Based on our third specification, which adds further controls for firm characteristics (firm size and innovativeness), Column (3) again shows an almost unchanged diffusion effect. The stepwise inclusion of potential confounders demonstrates that a strong diffusion effect arises even when we add many more potential confounders, i.e., the effect does not depend on a specific covariate selection.^19^

**Table 4:** Effect of curriculum updates on technology diffusion

Dependent variable	Technology use		
	(1)	(2)	(3)
Update	12.57***	10.50***	10.20***
	(2.87)	(2.91)	(2.95)
Constant	4.29	− 2.88	1.02
	(4.56)	(4.94)	(5.87)
R&D-stage diffusion	Yes	Yes	Yes
Channel	Yes	Yes	Yes
#AdWords	Yes	Yes	Yes
*Additional controls*			
Research papers	No	Yes	Yes
Firm size	No	No	Yes
Patent-applicant firm	No	No	Yes
Observations	4215	4215	4215
R-squared	0.089	0.135	0.137

Results from linear probability regressions, which pool event indicators before/after the respective curriculum update and use a single indicator capturing the diffusion effect (*Update*). *R&D-Stage Diffusion* refers to the index of cumulative patents (2019 as base year; squared and tripled terms included). *Channel* refers to the advertisement channel the job ad is posted in; *AdWords* corresponds the number of words in the job ad. *Research Papers* is an index of cumulative research papers (2019 as base year). *Firm Size* indicates whether the vacancy is at a small and medium-sized firm, while *Patent-Applicant Firm* captures whether the firm has filed any patent applications in the past. Authors’ calculations with data from the Swiss Job Market Monitor. Standard errors according to the complex survey design are reported in parentheses. Coefficients, standard errors, and sample means of the dependent vars are multiplied by 100 to represent percentage point changes

Significance levels: *p < 0.10, **p < 0.05, ***p < 0.01

Given these findings, we argue that curriculum updates generate a considerable diffusion advantage for those occupations updated earlier. Taking the average technology use in the pretreatment phase (11.59%) as a baseline, we argue that the updating effect corresponds to effectively doubling the baseline. Assuming an s-shaped diffusion curve and calculating the time in which technology use doubled before the updates (four years), we argue that the diffusion advantage broadly corresponds to an about four years faster technology diffusion.^20^ The diffusion advantage is crucial for productivity growth and firm competitiveness as previous studies show that earlier technology adoption has a large impact on such economic outcomes (Boothby et al., 2010; Crespi et al., 2008; Eaton & Kortum, 1999; Parente & Prescott, 1994; Skinner & Staiger, 2015).^21^

### Robustness, sensitivity, and validation tests

This subsection describes the additional tests that we conducted to examine the robustness and sensitivity of our main findings. We show that the diffusion effect materializes independently of the choice of our estimator and also whether we examine each technology separately in its own subsample or all three technologies at once. We further demonstrate that our findings are robust to using different functional forms for the estimation of the R&D-stage diffusion and show that excluding extreme latecomer occupations (i.e., with an update later than 2010) does not change our findings. Furthermore, we study whether increased investments in technologies (in this case, CNC machinery) accompanies curriculum updates. Finally, we explore how effects evolve when we extend our event study beyond 10 years after the updates. The following subsection explains these additional tests and their approaches in more detail.

First, as we use OLS to estimate the diffusion effect of curriculum updates and as our outcome variable (technology use) is binary, one question is how robust our results are to the use of non-linear estimator. Using probit and logit estimations, we find diffusion effects that are very similar to those with our main empirical approach, with effect sizes even a bit larger (see Tables 9 and 10 in the Appendix, which display the logit and probit results). We therefore argue that our overall empirical patterns are robust to different estimator choices.

Second, another potential concern lies with examining multiple technologies at once and pooling them into one sample. The average effect across all of the technologies could be driven by strong effects for only one of the technologies (absent effects for the other technologies), limiting the internal and external validity of our results. To examine whether our findings of a strong diffusion effect hold not only for the pooled sample of the three technologies but also within each of our technologies, we conduct subsample regressions. Our results show strong and significant diffusion effects within all three technologies (see Table 11 in the Appendix): after a curriculum update, the use of CAD on the job rises by 38 percentage points, of CNC by 10 percentage points, and of DP by 33 percentage points. These findings demonstrate that our main results are not driven by just one single technology but—instead—represent a generalizable diffusion effect of curriculum updates.^22^

Third, as R&D-stage diffusion acts as a vital input for the diffusion of new technologies into jobs, the choice of the underlying functional form of this relationship could also drive our results. To ensure that our results do not depend on a particular functional form, we estimate our event study using different functional forms for the relationship between R&D-stage diffusion and technology use (linear, quadratic, and cubic). Our results show that changing the functional form does not change the diffusion effect patterns (Appendix Table 12). In addition, we also take a fully nonparametric approach that corresponds to a generalized difference-in-differences estimation, with not yet treated occupations as the only control group. In this estimation diffusion varies freely over time, and two-year fixed effects capture technology trends.^23^ This approach yields roughly the same diffusion patterns (Appendix Table 13) as the event study specification that captures the natural diffusion trend according to the R&D-stage diffusion. This finding demonstrates that either directly modeling the natural trend according to the R&D-stage diffusion or indirectly inferring it from not yet treated occupations leads to the same results. Given these findings on different functional forms and from the fully nonparametric approach, we can rule out that specific modeling choices for the R&D-stage diffusion or the time trend drive our main results.

Fourth, as our technologies began entering the majority of the curricula in the late 1990s and early 2000s, one potential concern is that those occupations extremely lagging behind in their update might not be fully comparable to those occupations receiving the update early on. Four occupations (*Building Services Planner*, *Carpenter*, *Polydesigner*, and *Printing Technician*) are extreme latecomers, that is, they received the technology update after 2010. Excluding these latecomer occupations from our sample does not change our results. We find similar diffusion effects (see Table 14 in the Appendix). We therefore argue that our sample of occupations is adequately defined, that is, occupations with early and those with late updates are comparable. Occupations receiving their update late (i.e., after 2010) might be the consequence of an unfavorable position in the educational revision cycle. The last evaluation or revision of training material likely took place just before the new technologies became salient, leading to a rather long wait until the next revision cycle and update.

Fifth, if curriculum updates indeed accelerate technology diffusion into the workplace, then one would likely see that firms increase their investments in machinery and equipment related to new technology after the update. Particularly CNC technology requires specific expensive machinery that firms will only purchase, if they plan to use the new technology in the workplace. To examine whether firms actually increase their investments, we provide descriptive statistics on CNC imports into Switzerland. Our findings indicate that firms increasingly invest in complementary machinery at the same time at which the curriculum updates take place. However, given the descriptive nature of the data, we consider these findings as providing only supporting but not conclusive evidence. In particular, to capture firms’ investments in new CNC machinery, we draw on Comtrade data as an additional and external data source and focus on the two largest occupations that have introduced CNC: the Polymechanic and Automation Mechanic.^24^ These two occupations cover more than half of all apprenticeships in the CNC-treated occupations, involve an intensive use of CNC machinery in the workplace, and both received the CNC update in 1998.

In support of our main findings, we find that imports of CNC machinery into Switzerland appear to divert from the macro trend on imports after 1998 (see Appendix Figs. 8 and 9). We display the UK and the US as other industrialized countries that do not have vocational curriculum updates. As a small country, specific imports for Switzerland tend to fluctuate more than for the UK and US. Nonetheless, after 1998, CNC imports into Switzerland appear to start rising steadily—which is not the case for the US and the UK (see Appendix Fig. 8). We argue that rising CNC investments (captured through increasing imports), especially after the curriculum updates, further support the role of curriculum updates in accelerating technology diffusion in the workplace.

Sixth, one open question is how technology diffusion develops in the long run. Our results showed that curriculum updates accelerate technology diffusion in the short and medium run. Exploring how this accelerated diffusion evolves over a longer time horizon, we find that the diffusion advantage eventually tapers off because—given enough time—even mainstream firms (with or without updated curricula) learn how to use the new technologies and subsequently catch up (see Fig. 10). The diffusion advantage of occupations with early curriculum updates appears to last about 10 years. The fact that the diffusion effects are tapering off in the long run is also in line with findings from the technology diffusion literature, which shows that technology adoption takes the form of waves, at the end of which, technologies and skills become common knowledge across firms and industries and, eventually, almost all firms adopt the new technology (Baptista, 1999; Fuentelsaz et al., 2003; Jovanovic & Lach, 1989; Meade & Islam, 2006; Schlichte et al., 2019). Moreover, when widespread adoption is reached, firms have little incentive to mention technologies in job ads (as a way to better match jobs and job applicants) because the common use of these technologies is assumed and the next generation of technological innovation may need to be pointed out in the job ads.

### Further analysis: Curriculum updates as technology boost for mainstream firms?

Thus far, we have estimated the average diffusion effect across all firm types in our sample; however, the question arises as to how firms’ distance to the innovation frontier moderates the diffusion effect. Curriculum updates may have very different diffusion effects among frontier and mainstream firms. While frontier firms likely already possess innovative knowledge in-house and thus have lower technology adoption costs, mainstream firms lack the in-house capability to absorb the knowledge on new technologies and they do not possess the knowledge on how to train apprentices with newly required skills. These factors lead to higher total adoption costs for mainstream firms as compared to frontier firms.

We therefore argue that for mainstream firms an important feature of curriculum updates is the greater reduction in their adoption costs through the provision of ready-made training solutions for new skills (e.g., clearly defined training modules for apprentices; manuals and courses for instructors; or inter-company training courses, in which apprentices use novel machinery and technology that is not yet available in their training firm). Based on these factors, we expect mainstream firms to experience larger reductions in adoption costs and, consequently, to react more strongly to curriculum updates (compared to frontier firms, which already have lower adoption costs—even without the curriculum updates). In a further analysis, we therefore examine how the diffusion effect varies between job ads from mainstream firms and those from frontier firms.

We first need to identify the mainstream firms (and separate them from the frontier firms). According to our definition, mainstream firms differ from innovative firms (i.e., those with R&D efforts and patent applications) in that they are distant from the innovation frontier and do not actively drive technological innovation or possess the knowledge of the latest technological innovations. Moreover, mainstream firms are more likely to be found among small and medium-sized firms. These firms do not have the capacity to closely monitor technology trends and skill requirements, unlike large firms, which—even when they are not innovation drivers—still have the funds, the specialized personnel, and whole departments to monitor technology trends and skill requirements (Antonelli & Scellato, 2015; Vaona & Pianta, 2008). We therefore define small and medium-sized enterprises (SMEs) without patent applications as *mainstream firms*, a subgroup that accounts for 70% of the job ads in our sample, and the rest of the firms (large or with patent applications) as frontier firms.

To empirically separate mainstream from frontier firms, we draw on Google Patents Public Data (provided by IFI CLAIMS Patent Services), which provides information on the patent applications of Swiss firms. To match the firm names from the patent applications to the job ads, we use the legal form of the job-advertising entity (e.g., “Inc” or “LLC”) and identify company names within the job ads’ text. Our matching results yield percentages of firms with (and without) patenting activities that are very close to those found in representative Swiss innovation surveys.^25^ In addition, we use information from the SJMM on the size of the firms with the job vacancies. To identify the mainstream firms, we combine the patenting information with the firm-size information.

**Fig. 5:**
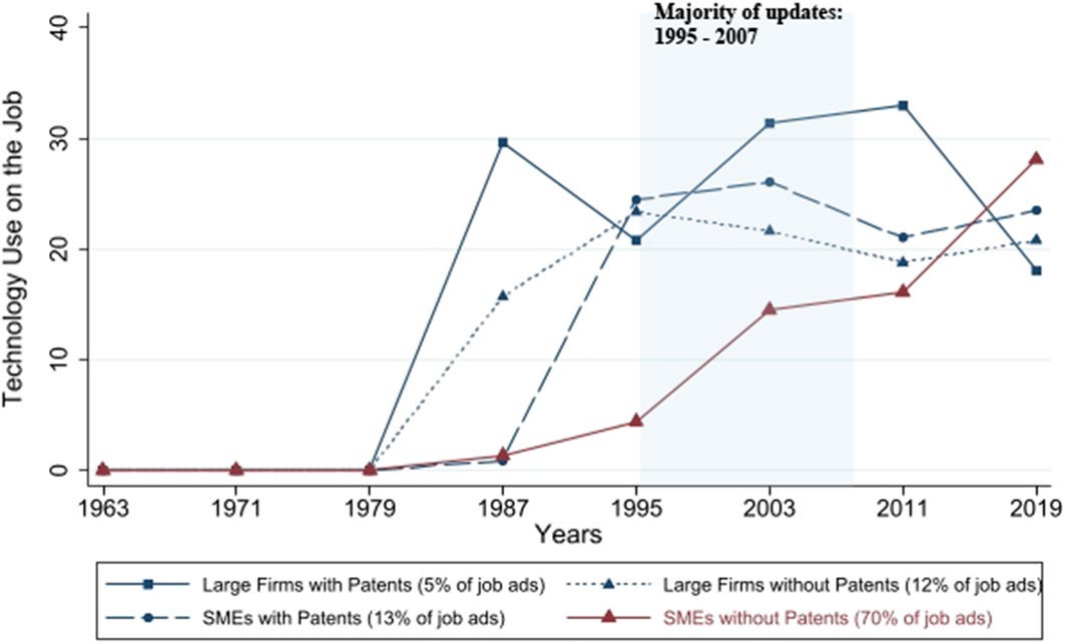
Technology diffusion by firm size and innovation status. *Notes* Average percentage of technology use across CAD, CNC, and DP and pooled in 8-year bins. The blue area captures the timeframe 1995–2007, in which more than 80% of our curriculum updates fall. Technology use multiplied by 100 to represent percentage points. Percentage of job ads refers to percentage of firm types in the sample of interest, i.e., job ads for drafter, mechanical engineering, and graphic designer occupations. Authors’ calculations with data from the SJMM.

**Fig. 6:**
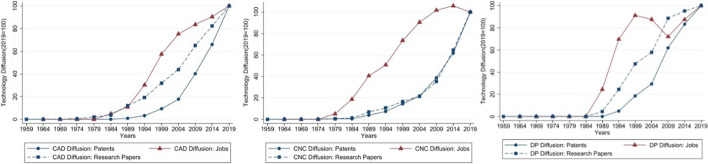
Technology diffusion curves for CAD, CNC, and DP. *Notes* Diffusion in patents refers to the index of cumulative patents for each technology in 5-year bins and uses 2015–2019 as base value, i.e., the 2015–2019 value is set to a 100. Diffusion into jobs is the average percentage of technology use in 5-year bins and as an index with 2015–2019 as base value. Diffusion in research papers refers to the index of cumulative research papers in 5-year bins and 2015–2019 as base value. Authors’ calculations with data from the SJMM

**Fig. 7:**
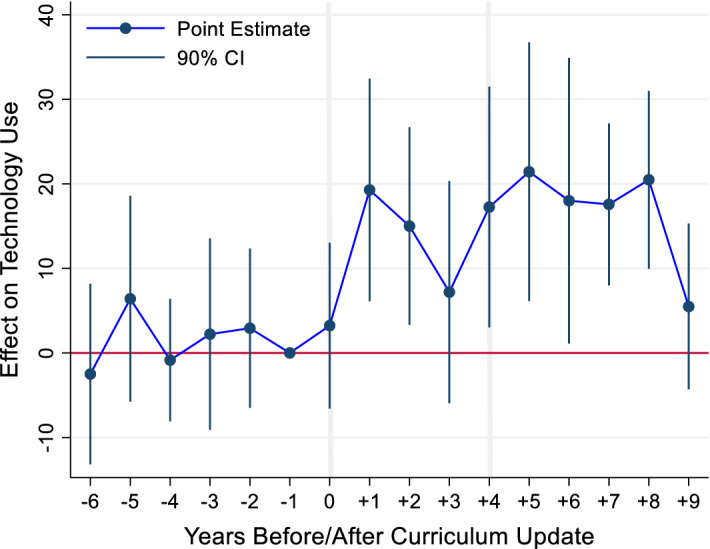
The effect of curriculum updates on technology use on the workplace (as measured by job ads, yearly event indicators). *Notes* Results from a linear probability regression using yearly event study indicators instead of pooling two years. Similar to our baseline specification, we control for R&D-stage diffusion, the number of words, and the advertisement channel of the job ad. Technology use multiplied by 100 to represent percentage points. The year before the enactment of a new curriculum serves as the reference category for the remaining event indicators and is therefore excluded. R&D-stage diffusion as an index with 2019 as base year; squared and tripled terms included. Standard errors based on the complex survey design by the SJMM.

**Fig. 10:**
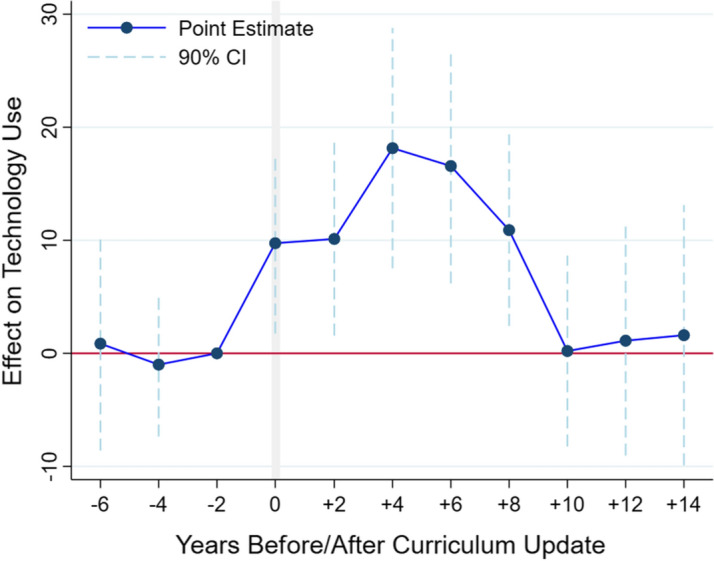
Long-run effects of curriculum updates on technology use (as measured by job ads). *Notes* Results from the linear probability regression, following our baseline specification, which controls for R&D-stage diffusion, the number of words and the advertisement channel of the job ad. To account for long-run effects, we included indicators for up to 16 years after the event. Technology use multiplied by 100 to represent percentage points. The two years before the enactment of a new curriculum serves as the reference category for the event indicators and are therefore excluded. R&D-stage diffusion as an index with 2019 as base year; squared and tripled terms included. Standard errors based on the complex survey design by the SJMM

Figure 5 shows the descriptive diffusion patterns for mainstream firms and compares these to the descriptive diffusion patterns for frontier firms. The mainstream firms exhibit diffusion patterns distinctly different from those found for the firms with better access to new technologies. The large firms with patents were the first to adopt the new technologies (these are likely also the firms that contributed innovative knowledge to the curriculum updating process), followed by the large firms without patents and the small firms with patents (which may often be startups). Compared to frontier firms, mainstream firms lagged behind in technology use in the 1980s and early 1990s. However, beginning with the period in which the majority of the curriculum updates fell (i.e., 1995–2007, with more than 80% of the updates taking place), technology diffusion strongly accelerated among the mainstream firms.

We account for the diffusion-pattern heterogeneity by examining how the status of a mainstream firm moderates the diffusion effect of an update. Our results in Table 5 show that, after an update, mainstream firms increase the use of new technologies more than frontier firms. For our baseline specification in Column 1, the diffusion effect is much larger for mainstream firms (15 percentage point increase, jointly significant at the 1% level) than for frontier firms. This finding of larger diffusion effects remains robust when we additionally controls for basic research in the second specification (Column 2). In sum, our results show that mainstream firms react more strongly to curriculum updates and accelerate technology adoption in the workplace more rapidly than frontier firms. Finding mainstream firms to be the most important drivers behind our key results makes a compelling case for the role of curriculum updates in helping firms and workers catch up to the innovation frontier and thereby reducing inequalities between firms, regions, and ultimately employees.

**Table 5:** Diffusion effect for mainstream firms

Dependent variable	Technology use	
	(1)	(2)
Update	5.60	3.64
	(5.60)	(5.62)
Update*Mainstream	9.40*	9.15*
	(5.53)	(5.22)
Mainstream	− 6.25	− 8.27*
	(4.40)	(4.31)
Constant	8.78	2.47
	(6.21)	(6.14)
R&D-stage diffusion	Yes	Yes
Channel	Yes	Yes
#AdWords	Yes	Yes
*Additional controls*		
Research papers	No	Yes
Observations	4215	4215
R-squared	0.09	0.14

Results from linear probability regressions, which pool event indicators before/after the respective curriculum update and use a single indicator capturing the diffusion effect (*Update*). *Mainstream* indicates whether the firm with the job ad is a mainstream firm, i.e., a small or medium-sized firm without patent applications. *R&D-Stage Diffusion* refers to the index of cumulative patents (2019 as base year; squared and tripled terms included). *Channel* refers to the advertisement channel the job ad is posted in; *AdWords* corresponds the number of words in the job ad. Research Papers is an index of cumulative research papers (2019 as base year). Firm size and patent-applicant status are excluded because they are already controlled for with the *Mainstream* indicator. Authors’ calculations with data from the Swiss Job Market Monitor. Standard errors according to the complex survey design are reported in parentheses. Coefficients, standard errors, and sample means of the dependent variables are multiplied by 100 to represent percentage point changes

Significance levels: *p < 0.10, **p < 0.05, ***p < 0.01

## Conclusion

In this paper, we demonstrated a direct link between the updating of education curricula with a particular technology skill and the increased use of that technology in firms’ workplaces. Focusing on CAD, CNC, and DP as three well-identifiable key technologies from an early wave of digitalization and controlling for R&D-stage diffusion (which captures the natural diffusion of technologies into jobs), we find a substantially accelerated diffusion after curriculum updates. After the implementation of an update and the start of apprenticeship training with the new technology skills, firms are on average about 12 percentage points more likely to use the new technology (measured by mentions of that particular technology in job ads for regular production jobs). This diffusion effect is in comparison with not-yet-updated occupations and controlled for R&D-stage diffusion.

Our findings show that curriculum updates substantially increase the speed at which new technologies diffuse into firms’ workplaces, that is, educational curriculum updates substantially shorten the time until new technologies arrive in the firms’ workplaces. After a curriculum update, firms become more aware of new technologies and can absorb the knowledge on new technologies faster. In particular, they can more easily complement the acquisition and introduction of new technologies with the necessary worker skills. Consistent with such mechanisms, we find that firms increase their investments in new equipment and machinery after the update (such as CNC machinery after the CNC update). As small delays in technology adoption can have a large impacts on economic outcomes (Comin & Hobijn, 2004; Eaton & Kortum, 1999; Giorcelli, 2019; Parente & Prescott, 1994), we argue that this accelerated diffusion likely contributes to productivity advantages and to an improved overall competitiveness as firms increasingly invest into and use new technologies.

Examining diffusion patterns more deeply with our event study design, we further show that the accelerated diffusion already occurs right after the legal enactment and during the early implementation phase of the new curriculum, that is, when the first apprentices undergo the updated training but have not even yet graduated. This effect likely occurs because, in the implementation phase, curriculum updates already strengthen firms’ absorptive capacity, heighten technological awareness, and also signal the arrival of new technology more broadly to the industry. Apprenticeship training consists of a combination of training in the workplace, vocational schools, and—most importantly—inter-firm training courses, which are often run by external (industry-wide) training centers. These external inter-firm training elements play an important role in the diffusion process, because they allow apprentices who may not have access to the new technologies in their own apprenticeship training company to actually gain hands-on experience in the new technologies externally and then bring these experiences into their training firms.

Furthermore, our event study findings show that the diffusion effect is strongest when the first apprentices graduate and become available as regular skilled workers on the internal or external labor market. The large share of Swiss apprentices switching firms after graduation (about 50%) likely explains why the diffusion effect is strongest right after the first apprentices graduate.

Examining the diffusion patterns more closely for the different types of firms, we uncover that mainstream firms (i.e., non-innovative SMEs) experience the strongest diffusion effects. These firms constitute the large majority of firms (96% of Swiss firms are non-innovative) and also post the majority of job ads in our sample (70% of job ads). These firms also make the greatest strides in catching up to the innovation frontier after an update: for these firms, the diffusion effect is with 15 percentage points much stronger than for more innovative or larger firms. This strong effect for mainstream firms relates to previous research in innovation economics and management science that points to the importance of using external knowledge sources as a diffusion mechanism (for the role of VET curricula in particular, see Rupietta & Backes-Gellner, 2019; for external knowledge in general, see Caiazza, 2016; Carlsson & Stankiewicz, 1991; Jovanovic & Lach, 1989; Lynn et al., 1996; Watkins et al., 2015a).

The findings of this paper have three broadly generalizable implications. First, our empirical evidence makes a compelling case for extending the perspectives on human capital investments beyond educational levels or years of education alone. Our results demonstrate the importance of examining not only enrollment within educational levels or educational types but also the quality and the up-to-datedness of the respective educational content. Taking these additional dimensions into account may enrich economic research with a more nuanced view of human capital investments. Especially in fast-changing economies with disruptive digital technologies, the quality and up-to-datedness of educational content will further gain in importance as critical factors for the labor market success of students and apprentices as well as for the competitiveness of firms and the economy as a whole. Our findings from earlier cycles of digital transformation suggest that systematic educational updates for middle-skill occupations can act as an effective diffusion device for bringing the latest digital technologies into firms’ workplaces, particularly into those of mainstream firms.

Second, and as a consequence of the above, our findings highlight the importance of studying and establishing mechanisms (such as legal frameworks, institutional structures or organizational procedures) that ensure the up-to-datedness of education curricula. The Swiss VET system, which covers the education and training of the entire middle-skill segment (two thirds of the working population), provides an example that is worth drawing from when designing such mechanisms. It offers inspiration for mechanisms that help to integrate new technologies into existing education programs (as opposed to creating new educational programs or encouraging additional years of education).

Third, updating educational programs at the occupational level may have broader benefits within the firms’ innovation ecosystems. Updating the training content of VET programs is likely particularly helpful for diffusing innovative knowledge on new technologies more broadly, because VET curricula can cover the training for the backbone of the workforce (i.e., almost all middle-skilled occupations) and across a large variety of different firms within the same and even across industries. A broader technology diffusion may increase the innovativeness of firms along the entire value chain of industries. Through participation in the updating process frontier firms can ensure the sufficient supply of future-oriented technical skills on the labor market and likely also enable a faster adoption of new technologies along the entire value chain. By affecting the technology adoption of firms both up- and downstream, curriculum updates may spur technology adoption more broadly across the entire innovation ecosystem (e.g., by suppliers also adopting the new technology and thereby strengthening the innovativeness of the entire industry).

This broad diffusion of new technologies likely goes hand-in-hand with the firm structure of the Swiss economy, which consists of many small- and medium-sized firms that are all relatively close to the technology frontier according to typical innovation indicators (Spescha & Wörter, 2018). The curriculum updating as part of the Swiss innovation system likely plays a key role in ensuring that even the small- and medium-sized firms can keep up with new technologies. In other developed economies (such as the US), some firms are at the leading edge of the technology frontier but also many other (particularly small and medium sized) firms struggle to keep up with the latest technological developments (Autor et al., 2020). Such economies may also benefit from better training middle-skilled workers and a broader technology diffusion, because broader technology adoption can create positive externalities along the value chain.

A fruitful avenue for future research will be to look more closely into the importance of the different channels through which curriculum updates strengthen the absorptive capacity of firms. While apprentices bring the ability to internalize the knowledge on new technologies into firms early on, it remains less clear which element of apprenticeship training plays the most critical role. One important element of Swiss apprenticeship training is inter-company training courses at training centers, where apprentices can get hands-on experience with new and expensive machinery, even if this machinery is not yet available at their training firm. Future research may examine more closely the importance of the different modes of training, i.e., the time spent in vocational schools, training in the workplace (formally or informally), or in inter-company training centers. Future research may illuminate the roles of each of these different elements for absorptive capacity and technology adoption. The findings may provide crucial information to policymakers on how to best structure vocational education and training programs and update them and also how to optimally allocate the available training time across the different elements.

## Figures and Tables

**Fig. 8:**
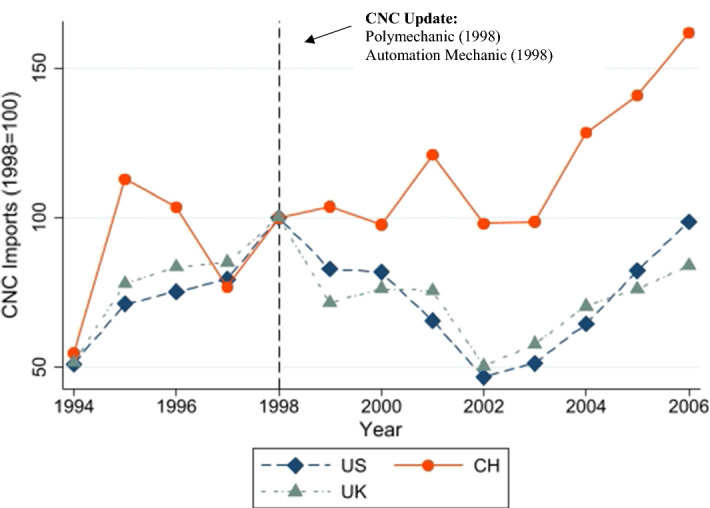
Imports of CNC machinery into Switzerland, the US, and the UK. *Notes* Value of imports of CNC-related machinery and parts as an index with 1998 as base year. Authors’ calculations with data from the Comtrade database

**Fig. 9:**
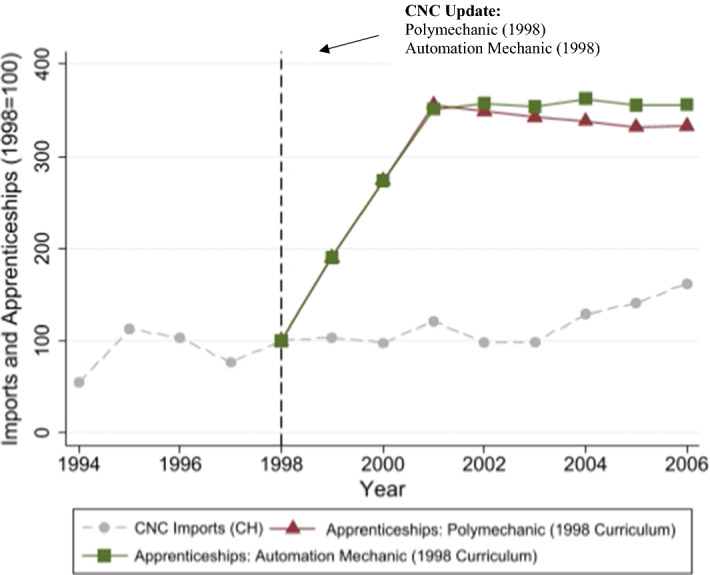
Imports of CNC machinery to switzerland and the number of ongoing apprenticeships. *Notes* Value of CNC-related machinery and parts imported to Switzerland as an index with base year 1998. Number of ongoing apprenticeships in the Polymechanic and Automation Mechanic occupations under the 1998 updated curriculum, as an index with base year 1998. Authors’ calculations with data from the Comtrade database and Datenbank Berufsentwicklung

## Appendix (Curriculum updating process: illustrative examples)

### Additional Tables and Figures

See Figs. 6, 7, 8, 9 and 10 and Tables 6, 7, 8, 9, 10, 11, 12, 13, 14, and 15.

**Table 6:** List of curriculum updates introducing new technologies (CAD, CNC, and DP)

Occupation	Year of update	Year of first graduates
*A. CAD introductions*		
Drafter, architecture (*Hochbauzeichner*)	1995	1999
Drafter, structure (*Tiefbauzeichner*)	1996	2000
Drafter, machinery (*Maschinenzeichner*)	1998	2002
Geomatician (*Geomatiker*)	1998	2002
Drafter, metal constructions (*Metallbauzeichner*)	1999	2003
Interior designer (*Innenausbauzeichner*)	1999	2003
Drafter, electrical systems (*Elektrozeichner*)	2000	2004
Drafter, spatial planning (*Raumplanungszeichner*)	2000	2004
Drafter, garden and landscapes (*Landschaftszeichner*)	2002	2006
Micro Designer (*Mikrozeichner*)	2002	2006
Building Services Planner (*Gebäudetechnikplaner*)	2010	2014
*B. CNC introductions*^a^		
Metal worker (*Metallbauer*)	1997	2001
Polymechanic (*Polymechaniker*)	1998	2002
Automation mechanic (*Automatiker*)	1998	2002
Production mechanic (*Decolleteur*)	2001	2005
Plant and equipment manufacturer (*Anlagenbauer*)	2002	2006
Micromechanic (*Mikromechaniker*)	2002	2006
Cabinet maker (*Schreiner*)	2002	2006
Carpenter (*Zimmermann*)	2014	2018
*C. DP introductions*		
Graphic designer (*Grafiker*)	2001	2005
Polygraph (*Polygraf*)	2002	2006
Mediamatician (*Mediamatiker*)	2003	2007
Interactive Media Designer (*Interactive Media Designer*)	2003	2007
Polydesigner (*Polydesigner*)	2010	2014
Printing Technician (*Drucktechnologe*)	2018	2022

CNC refers to computer-numerically controlled machinery, CAD to computer-aided design, and DP to desktop publishing software. *Year of Update* denotes the year in which the new curriculum went into effect. *Year of First Graduates* denotes the year when the first apprentices under the updated curriculum graduate

^a^Compared to the CNC introduction for the machining metal operators in Janssen and Mohrenweiser (2018) (for Germany), the introduction of CNC into the Swiss mechanical engineering occupations was relatively late. The difference likely relates to an updating round for the Swiss mechanical engineering occupations in the early and mid 1980s, when CNC technology was in an too-early stage for its inclusion into curricula. The next updating round did not start until the late 1990s

**Table 7:** Effect of curriculum updates on technology diffusion (Patent growth as additional control variable)

Dependent variable	Technology use			
	(1)	(2)	(3)	(4)
Update	10.53***	12.65***	11.54***	11.23***
	(2.46)	(2.86)	(2.96)	(3.01)
Constant	5.45	6.95	14.17*	18.62**
	(5.61)	(6.93)	(7.50)	(7.74)
*R&D-stage controls*				
Patent growth	Yes	Yes	Yes	Yes
R&D-stage diffusion	No	Yes	Yes	Yes
*Job Ad controls*				
Channel	Yes	Yes	Yes	Yes
#AdWords	Yes	Yes	Yes	Yes
*Additional controls*				
Research papers	No	No	Yes	Yes
Firm size	No	No	No	Yes
Patent-applicant firm	No	No	No	Yes
Observations	4215	4215	4215	4215
R-squared	0.09	0.09	0.14	0.14

Results from linear probability regressions, which pool event indicators before/after the respective curriculum update and use a single indicator capturing the diffusion effect (*Update*). *Patent Growth* refers to the relative growth in the stock of patents (year-over-year). *R&D-Stage Diffusion* refers to the index of cumulative patents (2019 as base year; squared and tripled terms included). *Channel* refers to the advertisement channel the job ad is posted in; *AdWords* corresponds the number of words in the job ad. *Research Papers* is an index of cumulative research papers (2019 as base year). *Firm Size* indicates whether the vacancy is at a small and medium-sized firm, while *Patent-Applicant Firm* captures whether the firm has filed any patent applications in the past. Authors’ calculations with data from the Swiss Job Market Monitor. Standard errors clustered at the occupational level are reported in parentheses. Coefficients, standard errors, and sample means of the dependent vars are multiplied by 100 to represent percentage point changes

Significance levels: **p* < 0.10, ***p* < 0.05, ****p* < 0.01

**Table 8:** Effect of curriculum updates on technology diffusion (Measured by technology keywords)

Dependent variable	#Technology keywords		
	(1)	(2)	(3)
Update	0.24***	0.19***	0.19***
	(0.05)	(0.05)	(0.05)
Constant	0.04	− 0.09	− 0.10
	(0.09)	(0.10)	(0.14)
R&D-stage diffusion	Yes	Yes	Yes
Channel	Yes	Yes	Yes
#AdWords	Yes	Yes	Yes
*Additional controls*			
Research papers	No	Yes	Yes
Firm size	No	No	Yes
Patent-applicant firm	No	No	Yes
Observations	4215	4215	4215
R-squared	0.10	0.13	0.13

Results from linear regressions, which pool event indicators before/after the respective curriculum update and use a single indicator capturing the diffusion effect (*Update*). The dependent variable is the number of technology keywords (i.e., CAD keywords for drafter occupations, CNC keywords for mechanical engineering and carpentry occupations, DP keywords for graphic design and media occupations). *R&D-Stage Diffusion* refers to the index of cumulative patents (2019 as base year; squared and tripled terms included). *Channel* refers to the advertisement channel the job ad is posted in; *AdWords* corresponds the number of words in the job ad. *Research Papers* is an index of cumulative research papers (2019 as base year). *Firm Size* indicates whether the vacancy is at a small and medium-sized firm, while *Patent-Applicant Firm* captures whether the firm has filed any patent applications in the past. Authors’ calculations with data from the Swiss Job Market Monitor. Standard errors according to the complex survey design are reported in parentheses

Significance levels: **p* < 0.10, ***p* < 0.05, ****p* < 0.01

**Table 9:** Effect of curriculum updates on technology diffusion (logit model, marginal effects)

Dependent variable	Technology use		
	(1)	(2)	(3)
Update	14.42**	14.57***	14.09***
	(5.79)	(5.25)	(5.12)
R&D-stage diffusion	Yes	Yes	Yes
Channel	Yes	Yes	Yes
#AdWords	Yes	Yes	Yes
*Additional controls*			
Research papers	No	Yes	Yes
Firm size	No	No	Yes
Patent-applicant firm	No	No	Yes
Pseudo R-squared	0.0934	0.155	0.158
Observations	4215	4215	4215

Results from logit models, which pool event indicators before/after the respective curriculum update and use a single indicator capturing the diffusion effect (*Update*). *R&D-Stage Diffusion* refers to the index of cumulative patents (2019 as base year; squared and tripled terms included). *Channel* refers to the advertisement channel the job ad is posted in; *AdWords* corresponds the number of words in the job ad. *Research Papers* is an index of cumulative research papers (2019 as base year). *Firm Size* indicates whether the vacancy is at a small and medium-sized firm, while *Patent-Applicant Firm* captures whether the firm has filed any patent applications in the past. Authors’ calculations with data from the Swiss Job Market Monitor. Standard errors according to the complex survey design are reported in parentheses. Coefficients, standard errors, and sample means of the dependent vars are multiplied by 100 to represent percentage point changes

Significance levels: **p* < 0.10, ***p* < 0.05, ****p* < 0.01

**Table 10:** Effect of curriculum updates on technology diffusion (Probit model, marginal effects)

Dependent variable	Technology use		
	(1)	(2)	(3)
Update	13.48**	14.16***	13.75***
	(5.50)	(4.95)	(4.89)
R&D-Stage Diffusion	Yes	Yes	Yes
Yes	Yes	Yes	Yes
#AdWords	Yes	Yes	Yes
*Additional controls*			
Research papers	No	Yes	Yes
Firm Size	No	No	Yes
Patent-applicant firm	No	No	Yes
Pseudo R-squared	0.095	0.143	0.145
Observations	4215	4215	4215

Notes: Results from probit models, which pool event indicators before/after the respective curriculum update and use a single indicator capturing the diffusion effect (*Update*). *R&D-Stage Diffusion* refers to the index of cumulative patents (2019 as base year; squared and tripled terms included). *Channel* refers to the advertisement channel the job ad is posted in; *AdWords* corresponds the number of words in the job ad. *Research Papers* is an index of cumulative research papers (2019 as base year). *Firm Size* indicates whether the vacancy is at a small and medium-sized firm, while *Patent-Applicant Firm* captures whether the firm has filed any patent applications in the past. Authors’ calculations with data from the Swiss Job Market Monitor. Standard errors according to the complex survey design are reported in parentheses. Coefficients, standard errors, and sample means of the dependent vars are multiplied by 100 to represent percentage point changes

Significance levels: **p* < 0.10, ***p* < 0.05, ****p* < 0.01

**Table 11:** Effect of curriculum updates on use of CAD, CNC, and DP

Dependent variable	(1) CAD use	(2) CNC use	(3) DP use
Update	37.86^***^	9.52^***^	32.71^***^
	(7.38)	(2.87)	(11.01)
Constant	4.14	4.34	1.50
	(2.98)	(4.85)	(3.34)
R&D-stage diffusion	Yes	Yes	Yes
Channel	Yes	Yes	Yes
#AdWords	Yes	Yes	Yes
Observations	804	3223	188
R-squared	0.306	0.036	0.196

Results from linear probability regressions, which pool event indicators before/after the respective curriculum update and use a single indicator capturing the diffusion effect (*Update*). *CAD Use*, *CNC Use*, and *DP Use* refer to the use of the respective technology. *Update* denotes whether the job ads were posted before or after the update. *R&D-Stage Diffusion* refers to the index of cumulative patents (2019 as base year; squared and tripled terms included). *Channel* refers to the advertisement channel the job ad is posted in. *AdWords* corresponds the number of words in the job ad. *Research Papers* is an index of cumulative research papers (2019 as base year). Authors’ calculations with data from the Swiss Job Market Monitor. Standard errors according to the complex survey design are reported in parentheses. Coefficients, standard errors, and sample means of the dep. var. are multiplied by 100 to represent percentage point changes

Significance levels: **p* < 0.10, ***p* < 0.05, ****p* < 0.01

**Table 12:** Effect of curriculum updates: different functional forms of R&D diffusion

Dependent variable	Technology use		
	(1)	(2)	(3)
	Linear R&D-diffusion model	Quadratic R&D-diffusion model	Cubic R&D-diffusion model
T_Update_ − 7 or earlier	− 1.23	− 6.02	− 9.13^*^
	(4.56)	(5.18)	(5.28)
T_Update_ − 6	2.77	1.49	0.70
	(5.93)	(5.79)	(5.71)
T_Update_ − 4	− 0.73	− 0.97	− 0.97
	(3.91)	(3.85)	(3.87)
T_Update_	8.88^*^	9.60^**^	9.74^**^
	(5.08)	(4.86)	(4.85)
T_Update_ + 2	9.46^*^	9.82^*^	10.11^*^
	(5.28)	(5.19)	(5.18)
T_Update_ + 4	16.16^**^	17.54^***^	17.97^***^
	(6.68)	(6.47)	(6.49)
T_Update_ + 6	13.88^**^	15.57^**^	16.39^***^
	(6.31)	(6.25)	(6.33)
T_Update_ + 8	7.64	9.94^*^	11.00^**^
	(5.09)	(5.09)	(5.11)
T_Update_ + 9 or later	− 0.49	2.05	2.01
	(5.81)	(5.88)	(5.85)
*R&D-stage diffusion*			
Patents	15.35	− 24.05	− 70.23^*^
	(10.47)	(22.21)	(38.71)
Patents^2^		31.53^*^	141.21
		(18.35)	(99.69)
Patents^3^			− 68.80
			(67.20)
Channel	Yes	Yes	Yes
#AdWords	Yes	Yes	Yes
Constant	2.44	18.60^**^	21.77^***^
	(4.55)	(7.28)	(7.58)
Observations	4215	4215	4215
R-squared	0.098	0.100	0.100

The event indicators cover two years. *T*_*Update*_ corresponds to the year in which the respective curriculum update was enacted and the year after. The reference category are the two years before the update (T_Update_ – 2) and therefore omitted. *R&D-Stage Diffusion* refers to the index of cumulative patents (2019 as base year; squared and tripled terms included). *Channel* refers to the advertisement channel the job ad is posted in. *AdWords* corresponds the number of words in the job ad. Standard errors according to the complex survey design are reported in parentheses. Coefficients, standard errors, and sample means of the dep. var. are multiplied by 100 to represent percentage point changes

Significance levels: **p* < 0.10, ***p* < 0.05, ****p* < 0.01

**Table 13:** Effect of curriculum updates: non-parametric diffusion trend

Dependent variable	CAD use	(2) CNC use	(3) DP use
Update	28.97^***^	8.44^***^	21.64^**^
	(7.64)	(1.91)	(10.38)
Constant	23.88^**^	0.39	15.97
	(10.12)	(3.64)	(12.42)
Two-year fixed effects	Yes	Yes	Yes
Channel	Yes	Yes	Yes
#AdWords	Yes	Yes	Yes
Observations	804	3,223	188
R-squared	0.333	0.049	0.310

**Table 14:** Effect of curriculum updates on technology diffusion (no latecomers)

Dependent variable	Technology use		
	(1)	(2)	(3)
Update	10.31**	14.30***	13.85***
	(4.37)	(4.64)	(4.61)
Constant	6.86	− 0.34	5.62
	(4.98)	(5.25)	(7.22)
R&D-stage diffusion	Yes	Yes	Yes
Channel	Yes	Yes	Yes
#AdWords	Yes	Yes	Yes
*Additional controls*			
Research papers	No	Yes	Yes
Firm size	No	No	Yes
Patent-applicant firm	No	No	Yes
Observations	3745	3745	3745
R-squared	0.08	0.11	0.12

**Table 15:** Effect of curriculum updates on technology diffusion (Standard errors clustered at occupational level)

Dependent variable	Technology use		
	(1)	(2)	(3)
Update	12.57**	10.50**	10.20**
	(4.83)	(4.92)	(4.86)
Constant	7.51	− 0.90	3.96
	(4.65)	(6.16)	(7.88)
R&D-stage diffusion	Yes	Yes	Yes
Channel	Yes	Yes	Yes
#AdWords	Yes	Yes	Yes
*Additional controls*			
Research papers	No	Yes	Yes
Firm size	No	No	Yes
Patent-applicant firm	No	No	Yes
Observations	4,215	4,215	4215
R-squared	0.089	0.135	0.137

Results from linear probability regressions, which pool event indicators before/after the respective curriculum update and use a single indicator capturing the diffusion effect (*Update*). *R&D-Stage Diffusion* refers to the index of cumulative patents (2019 as base year; squared and tripled terms included). *Channel* refers to the advertisement channel the job ad is posted in; *AdWords* corresponds the number of words in the job ad. *Research Papers* is an index of cumulative research papers (2019 as base year). *Firm Size* indicates whether the vacancy is at a small and medium-sized firm, while *Patent-Applicant Firm* captures whether the firm has filed any patent applications in the past. Authors’ calculations with data from the Swiss Job Market Monitor. Standard errors clustered at the occupational level are reported in parentheses. Coefficients, standard errors, and sample means of the dependent vars are multiplied by 100 to represent percentage point changes

Significance levels: **p* < 0.10, ***p* < 0.05, ****p* < 0.01

### Updating VET curricula: cases from the mechanical engineering and dental industries

In the following, we provide additional information on the curriculum updating processes in the Swiss VET system and explain how new technologies are systematically integrated into updates. We illustrate with case studies and anecdotal evidence from the mechanical engineering and dental industries (based on Backes-Gellner & Pfister, 2019), how—in practice—new technologies find their way into curricula and how strongly innovative firms are involved in the updating process.

Both the mechanical engineering and the dental industry have been facing important new technologies in the last decades. In mechanical engineering, since the early 1990s computer-controlled machine tools, automation, and programmable logic controllers emerged and became important for the work of mechanical engineers (“mechanics”)^26^; in dentistry, in the early 2010s, new digital workflow technologies gained momentum among dental technicians. As the updating of occupational curricula occurs regularly (as prescribed by law), in both industries these new technology trends were spotted at an early stage with the help of the most innovative firms. With evaluation and updating of curricula scheduled, the industry organizations conducted broad surveys among firms and gathered information on new technologies, future occupational activities, and new skill requirements from their members, including the most innovative firms.

Using these survey results, occupational expert groups—consisting largely of those delegated from firms (often very innovative firms)—developed new task profiles for the affected occupations of the industries. Through these expert groups, the knowledge of frontier firms entered into the curriculum updating process. For example, for the mechanical engineering industry, frontier firms and their experts contributed in the 2009 update (in a very early stage) specific knowledge on the use of programmable logic controllers (which is an integrated hard- and software combination for production control) and on the methods for training the required skills. For the dental industry, as another example, experts from innovative laboratories that had already embraced the digital transformation contributed in the 2018 update their digital expertise on digital scanning, rendering, and 3D-printing (and a new type of digital workflow) to the new occupational profile.

In both industries firms discussed and, in a second step, together with the professional associations and legal authorities agreed on the new occupational profiles. For the mechanical engineering industry, more knowledge of programmable logic controllers became part of the training curriculum for mechanics. In the dental industry, the training in digital workflows, with 3D-scanners, and 3D-printing technologies became part of the new curriculum for dental technicians. Moreover, inter-firm training courses were designed for teaching skills and providing hands-on experience for apprentices from companies that had not yet used the new technologies in their own production processes. After the update and legal implementation, the new curricula became mandatory for all firms training apprentices in the respective occupations. Thus, even apprentices in mainstream firms acquired the skills for frontier technologies, which allowed them to be ahead (in their skills) of their training companies’ technology.

The cases from the dental and mechanical engineering industries show how—in the updating process—frontier firms contribute their local frontier knowledge about which technologies they expect to be important and which skills they consider to be vital for apprentices in the upcoming years. Curriculum updates act as diffusion devices that make knowledge on new technologies, on the required skills, and on the methods of training them accessible to mainstream firms. The greater availability of both knowledge and skills to mainstream firms fosters the adoption of new technologies and brings these technologies into faster use in the workplace.

## Footnotes

1 Switzerland has the largest share of work-based education among the OECD countries (Hoeckel et al., 2009).

2 Other case studies on the innovativeness of the VET curriculum development system are, for example, Pedró et al. (2009).

3 The currently applying rules for the updating process (such as time intervals) are codified in the Swiss Handbook for Occupational Development Processes (State Secretariat for Education, Research and Innovation, 2017). Currently the best practice is that curricula are scheduled for an evaluation of the up-to-datedness of content on a regular basis of approximately 5 years.

4 The occupations in our sample only merge with other occupations into new occupations (no splits). We track the occupations over their entire evolution, taking all predecessor and successor occupations into consideration.

5 Our event study approach exploits different timings of curriculum updates for different Swiss VET occupations from different industries. This approach ensures that if we find jumps and discontinuities in technology use right after an update, that these jumps are likely caused by the curriculum updates themselves irrespective of the natural technology diffusion, which might move faster (or slower) in Switzerland compared to other countries. If diffusion were generally faster in Switzerland, then we would not find such discontinuities.

6 As occupations are highly standardized, applicants for jobs with a certain occupational requirement are expected to match this requirement with a formal training and a VET diploma in the stated occupation.

7 One potential concern is that by including R&D jobs we will also measure investments in developing new technologies instead of measuring actual technology diffusion.

8 In the SJMM, job ads with R&D tasks are those which are classified under the main tasks of “analyzing, researching, and monitoring”.

9 Based the occupation advertised in the job ad, we determine the technology that the job potentially might use, i.e., CAD for drafter occupations, CNC for engineering and carpentry occupations, and DP for design and media occupations.

10 One potential concern is that the use of different technologies overlaps within jobs, i.e., that a job ad refers to more than one technology. However, we find very little overlap (32 observations for CAD and CNC and 21 observations for CAD and DP out of 4215 observations). Neither assigning jobs to a particular technology nor taking a pooled approach changes our results in a meaningful way.

11 Each job ad is assigned to the occupation that the firm is looking for and, based on the occupational group, to one specific technology group.

12 Recent literature highlights the issues of forbidden comparisons in difference-in-difference settings with staggered treatments (Borusyak et al., 2021; Callaway & Sant’Anna, 2021; Chaisemartin & d'Haultfoeuille, 2020; Roth et al., 2022). Given our data constraints, we aim to indirectly tackle these issues through a parametric approach that models the counterfactual (Krupp & Pollard, 1996; Siming, 2017).

13 An alternative event-study approach consists of inferring the natural diffusion trend solely by the technology use in not-yet treated occupations through technology-specific time fixed effects. Given the peculiarities of our data such as the asymmetric distribution of observations across occupations and the need for modeling technology-specific time trends, this alternative approach would likely lead to overfitting our model when combining both event-study indicators and technology-specific time trends. In our main analysis, we therefore rely on the R&D diffusion; as robustness check, we take also the second approach with time fixed effects and find similar results.

14 As an additional check we also estimate regressions with yearly event indicators, which show similar diffusion patterns.

15 We bin job ads more than six years before the event and more than 10 years after the event into two additional separate event indicators.

16 The hiring of workers as instructors likely plays only a minor role for technology diffusion compared to the above mentioned diffusion channels. Apprenticeship instructors are in general long-term employees at the training companies, who in addition to a successful work history underwent extra training and passed an official examination to become an apprenticeship instructor. Whenever curricula are updated and require the teaching of new skills, all instructors are required to acquire these new skills and learn how to teach them. Thus, an important element of the official curriculum updating process are instructor courses and the provision of teaching material for instructors, because instructors use such material to learn on how to best train the newly added skills. However, the hiring of external workers (that already possess the newly required skills) to turn them into instructors is unlikely, because such workers would need to pass extra training and instructor examinations first before being able to train apprentices. Thus, we do not expect the hiring as instructors to be a substantial part of the diffusion effect that we observe from job ads.

17 As the SJMM represents the Swiss job market for regular workers, not for apprenticeships, the increase in technology use applies to regular jobs that target skilled workers with VET diplomas. Moreover, this increase occurs before the first apprentices graduate under the updated curriculum. The mentions of technologies thus do not come from an availability effect at the apprenticeship level (i.e., firms mentioning technology skills in the job ads to reflect the updated curriculum and the new skills of apprentices) but from an actual increase in technology use in the regular workers’ jobs.

18 As recommended by the SJMM, for all our calculations we account for the complex survey design behind the data set. The SJMM employs a stratification of newspaper media depending on region and circulation, website media depending on sectors and size of the firm, and job portal media (exhaustive sampling) depending on job categories. Selected advertising media from these strata serve as primary sampling units (PSU). We take the specifics of the PSUs into account and weight observations according to the probability of choosing the advertisement medium in which the job ad was published and selecting this job ad within the medium (two-stage cluster sampling). We use a Taylor-linearized variance estimation, also taking the complex survey design into account for the calculation of standard errors. Alternative weighting and clustering approaches (such as simple probability weighting and clustering standard errors at the occupational level) yield similar results (see, Table 15).

19 Moreover, in extra specifications, we also explore the role of patent growth (as speed of R&D-stage diffusion) as another independent variable and find similar results of technology diffusion (see, Table 7 in the Appendix). In addition to examining different selections of independent variables, we have also studied alternative outcome variables, e.g., the number of technology keywords in job ads. Using the number of technology keywords in job ads as an alternative indicator for technology diffusion, we also find substantial effects of the curriculum updates on this outcome (see Table 8 in the Appendix). If we interpret the number of technology keywords as capturing also the intensive margin of technology diffusion, i.e., the extent to which jobs use new technologies (as opposed to only whether jobs use new technology or not), we arrive at even slightly larger diffusion effects (compared to only examining effects along the extensive margin).

20 Previous studies find lags of several years in technology adoption across countries and between different types of firms (Atkin et al., 2017; Comin & Hobijn, 2010; Sanders & Tuschke, 2007).

21 Spreading new technologies into the middle-skill segment of VET occupations also enables more efficient production processes because the decreased reliance on high-skill workers allows for more efficient and flexible production processes, e.g., when middle-skilled workers program CNC machines in-house (Backes-Gellner, 1996; Ewers et al., 1990).

22 The differences in effect size can be explained by the diversity of tasks in the occupational groups using the technologies. For example, mechanical engineering occupations are very diverse in their tasks and make up for almost two-thirds of our sample. For these occupations, tasks with CNC machinery are one task type among many others. The task diversity likely dilutes the diffusion effect. In contrast, design and media occupations compose a small group of occupations that are very narrow in their task profiles. Almost all of them use DP technologies for the majority of tasks after the update.

23 Adding technology-specific yearly time trends to our model would mean adding 40 years of indicator variables for each of our three technologies (120 indicator variables in total). To avoid overfitting our model (given the sample size), we use two-year fixed effects and pool effects before and after the curriculum updates. As each technology follows its own trend, we use technology subsamples and report results separately for each technology.

24 Comtrade data is provided by the United Nations Statistics Division.

25 In the entire SJMM sample (1950–2019), we matched approximately 11.8% of all of the job ads to firms with patent applications. Taking into account that larger firms and growing innovative firms post more job ads, we find these numbers to be very close to those in the KOF Innovation Survey, which finds that in our observation period 4% of Swiss firms recently applied for a patent (Arvanitis et al., 2013; Spescha & Wörter, 2018). For a more accurate comparison, we also examined the percentages of patent applicants within the firm-size categories: for large firms, we matched 20.9% of job ads to patent applicants—a number also close to the 27% of large firms with recent patent applications in the KOF Innovation Surveys (Spescha & Wörter, 2018).
